# A Numerical and Experimental Investigation of the Most Fundamental Time-Domain Input–Output System Identification Methods for the Normal Modal Analysis of Flexible Structures

**DOI:** 10.3390/s25041259

**Published:** 2025-02-19

**Authors:** Şefika İpek Lök, Carmine Maria Pappalardo, Rosario La Regina, Domenico Guida

**Affiliations:** 1 Defense Industries Research and Development Institute, Scientific and Technological Research Council of Türkiye (TÜBİTAK), 06105 Ankara, Türkiye; sefika.lok@tubitak.gov.tr; 2 Department of Industrial Engineering, University of Salerno, Via Giovanni Paolo II 132, 84084 Fisciano, SA, Italy; rlaregina@unisa.it (R.L.R.); guida@unisa.it (D.G.)

**Keywords:** structural dynamics, mechanical vibrations, mechanical systems diagnostics, time-domain system identification methods, experimental modal analysis, vibrating structures

## Abstract

This paper deals with developing a comparative study of the principal time-domain system identification methods suitable for performing an experimental modal analysis of structural systems. To this end, this work focuses first on analyzing and reviewing the mathematical background concerning the analytical methods and the computational algorithms of interest for this study. The methods considered in the paper are referred to as the AutoRegressive eXogenous (ARX) method, the State-Space ESTimation (SSEST) method, the Numerical Algorithm for Subspace State-Space System Identification (N4SID), the Eigensystem Realization Algorithm (ERA) combined with the Observer/Kalman Filter Identification (OKID) method, and the Transfer Function ESTimation (TFEST) method. Starting from the identified models estimated through the methodologies reported in the paper, a set of second-order configuration-space dynamical models of the structural system of interest can also be determined by employing an estimation method for the Mass, Stiffness, and Damping (MSD) matrices. Furthermore, in practical applications, the correct estimation of the damping matrix is severely hampered by noise that corrupts the input and output measurements. To address this problem, in this paper, the identification of the damping matrix is improved by employing the Proportional Damping Coefficient (PDC) identification method, which is based on the use of the identified set of natural frequencies and damping ratios found for the case study analyzed in the paper. This work also revisits the critical aspects and pitfalls related to using the Model Order Reduction (MOR) approach combined with the Balanced Truncation Method (BTM) to reduce the dimensions of the identified state-space models. Finally, this work analyzes the performance of all the fundamental system identification methods mentioned before when applied to the experimental modal analysis of flexible structures. This is achieved by carrying out an experimental campaign based on the use of a vibrating test rig, which serves as a demonstrative example of a typical structural system. The complete set of experimental results found in this investigation is reported in the appendix of the paper.

## 1. Introduction

### 1.1. Formulation of the Problem of Interest for This Study

This paper deals with the principal time-domain system identification methods and their application to the experimental modal analysis of flexible structures [1,2]. Generally, there are four fundamental steps in the practical implementation of system identification methods [3,4,5], as discussed in detail below.

First, an experimental system is built, and input–output signals are measured from the experimental system [6]. Even if all the system identification computational approaches are based on sound mathematical grounds, the success of the identification process varies depending on the specific set of data used [7,8,9]. For this reason, before determining the model structure suitable for the dataset and using it to estimate the mathematical model of the system of interest, it is necessary to construct a suitable test rig with a proper set of sensors and actuators, leading to large signal-to-noise data records [10,11].

The second step is to determine the model and the method to be used to estimate the mathematical model of the system of interest [12]. Many methods are used in the system identification process. These methods can be classified as time-domain techniques or frequency-domain techniques depending on the general approach employed [13,14], discrete-time models or continuous-time models with respect to the time variable [15,16], and linear-based systems or nonlinear-based systems with respect to the model mathematical structure [17,18]. From a general perspective, it is challenging to establish which method is the most successful. Therefore, it is essential to be able to compare the performance of different system identification methodologies when applied to the same case study, especially in the case of the analysis of dynamic systems of engineering interest, such as the flexible structures typical of mechanical and civil engineering applications, and this is one of the main motivations behind the development of the present paper.

Third, another critical step is represented by the choice of the model parameters to be determined through the identification process [19]. Typically, the model parameters are chosen according to general criteria such as the Akaike information criterion (AIC), the final prediction error (FPE) criterion, and the minimum description length (MDL) criterion, which serve to optimize the number and the type of model parameters, as well as to increase the likelihood of success of the identification process [20,21,22,23].

Finally, the last step to be considered in this vein is the validation of the identified models [24]. For this purpose, identification and validation datasets that significantly differ from each other are used in this last step of implementing the chosen system identification method. In particular, the identification dataset is used to estimate the mathematical model of the system. In contrast, the validation dataset is used to test whether the estimated mathematical model memorizes the dataset or estimates the system successfully [25,26,27,28]. As discussed in the paper, the correct implementation of the four fundamental steps mentioned before considerably contributes to the success of the system identification process.

This paper proposes a comparative analysis, performed numerically and experimentally, of the most fundamental system identification methods found in the literature and poses the basis for developing a new family of time-domain input–output identification algorithms. The primary focus is using these methods to conduct experimental modal analysis of flexible structures. Compared to existing research, the novel aspects and advantages of this work lie in creating a seamless approach for constructing first-order state-space models and second-order configuration-space models of mechanical systems from vibration measurements and the acquisition of excitation signals.

### 1.2. Literature Review

This subsection reviews a substantial body of relevant literature, encompassing the applications of system identification methods for solving various challenging problems in the field of mechanical engineering, as discussed below.

Numerical procedures based on system identification methods are widely used to estimate mathematical models of mechanical systems, beam structures, biomechanical systems, space structures, and robotic manipulators [29,30,31]. In the literature, traditional system identification methods, such as the ARX, ARMAX, and output error methods, and subspace system identification methods are commonly employed to identify reduced-order models of mechanical systems [32,33,34,35]. A short literature survey about the issues of interest for this study is reported in the paper. More specifically, a brief literature survey on the practical use and the engineering applications of system identification methods developed in the field of mechanical engineering is provided below.

In a series of recent works [36,37,38], Peeters et al. proposed a frequency-domain identification method referred to as the PolyMAX method, namely, the poly-reference least-squares complex frequency-domain method, since this algorithm is based on a weighted least-squares approach and uses multiple-input multiple-output frequency response functions as input data. This method can be implemented similarly to the time-domain numerical procedures of interest for this investigation [39,40]. In [41], O’Higgins et al. proposed a minimal information data modeling (MID) method for performing structural health monitoring (SHM) of a short-span bridge. Additionally, O’Higgins et al. proposed, in [42], a technique to maximize the information obtained from low-signal-to-noise accelerometers used for durability analyses of large bridges. In [43], Yuan et al. proposed an improved method based on the unscented Kalman filter (UKF) approach for synchronously identifying nonlinear parameters of structural systems subjected to unknown excitations. In [44], Dong et al. proposed an extensive review of the parametric system identification methods based on the use of neural networks, including a discussion on the advantages and limitations of this approach. Subramanian et al. identified a full-tank system from first principles and with the prediction error, and N4SID methods. They demonstrated the superiority of the N4SID method with different input signals [45]. Borjas et al. investigated and compared four subspace identification techniques, the N4SID, IV-4SID, MOESP, and CVA methodologies, for ten industrial processes [46]. Juriek et al. compared various identification techniques for modeling a MIMO system and observed that the MOESP and CVA approaches had the most successful results [47]. Mola et al. proposed a novel identification technique for nonlinear system identification and used it to identify a flexible robot arm [48]. In [49], the authors worked on vibration analysis of a civic tower at Rieti using the OKID method to identify the first five mode shapes of the tower. Simay et al. compared the most used subspace identification approaches, the N4SID, CVA, and MOESP methods, using fifteen publicly available experimental datasets [50]. Hereditia et al. applied the ERA/OKID identification algorithm to real helicopter flight data for sensor failure detection of a small autonomous helicopter [51]. Guida et al. tested the ERA/OKID identification method to identify the parameters of a linear mechanical system on a lightly damped mechanical apparatus [52].

Jirasek et al. studied the dynamic behavior of a roof prototype constructed for an active hybrid structure employing the linear parameter-varying (LPV) framework [53]. Chang et al. worked on developing the OKID subspace identification technique and compared it with the ARX and N4SID techniques [54]. In [55], Qin presented an overview of substantial identification techniques for open-loop and closed-loop systems. Wang et al. proposed a novel identification algorithm based on the OKID technique to estimate unknown dynamic systems [56]. Skolnik et al. studied to estimate structural frequencies, damping rates, and mode shapes of the first nine modes for a fifteen-story steel structure with a real-earthquake dataset and used a finite element model of the building to improve the data correlation [57]. Qidwai et al. investigated a substantial identification technique for metal defect detection and tested it with actual ultrasonic vibrations [58]. In [59], subspace identification methods such as the MOESP, N4SID, and ORT methods and their variations were compared using chemical process datasets. Tuhta and Gunday used the N4SID method to estimate the state-space model of an industrial building [60]. The authors used the same method to identify an RC building [61]. Gautier et al. presented a new subspace fitting method that obtains the rigidity of an experimental rotating machine by minimizing the correlation between the theoretical observability matrix and the experimental observability matrix estimated using the MOESP method [62].

Mazzeo et al. proposed a method based on the empirical Fourier decomposition (EFD) technique to automatically achieve modal parameter identification of deformable structures from their free-vibration response [63]. To obtain physical parameters such as the mass, stiffness, and damping coefficients from state-space models of mechanical systems, a novel methodology was presented in a series of research papers [64,65,66,67]. In this vein, Angeles et al. presented a novel approach that requires only one sensor or one actuator at all degrees of freedom to obtain the desired physical parameters [68]. The authors of the present paper also tried improving the proportional damping coefficients of structural systems by using the estimated natural frequencies and the estimated damping ratios with a simple least-squares estimation approach [69]. In [70], Malgaca and Uyar analyzed a flexible composite manipulator and experimentally developed a hybrid vibration controller with piezoelectric actuators. In [71], Malgaca et al. identified the continuous-time transfer function of a flexible manipulator starting from experimental data. Koc et al. analyzed the vibration mode shapes of a spinning annular disk considering diverse boundary conditions [72]. Tufekci et al. studied the forced dynamical behavior of a rotating disk by using the Galerkin method [73]. Li et al. developed an identification method for a full-size rotor system and verified the technique proposed in their paper by numerical and experimental tests [74].

In [75], Peng et al. proposed a crowdsensing approach to indirectly identify the mode shapes of a bridge from the vibration responses of vehicles moving on it. Recently, a methodology was proposed in [76] to identify the modal damping ratio of mechanical systems, and the effectiveness of the proposed method was evaluated numerically with a five-degrees-of-freedom system and experimentally with a two-degrees-of-freedom mechanical system. Yokoyama studied a curve fitting approach using a minimum error criterion to identify the damping ratio of a single-degree-of-freedom spring–mass–damper system [77]. Pirrotta and Russotto identified natural frequencies, damping ratios, and modal shapes of a three-story frame system using the operational modal analysis method [78]. In [79], Lok et al. developed an active vibration controller for a single-link composite box manipulator employing system identification techniques, the proportional–derivative control strategy, and positive position feedback. Uyar et al. used time-domain system identification methods to design a vibration controller for a flexible manipulator [80]. In [81], Sands proposed a new computational approach for mathematical system identification based on feedforward and feedback controls. Per et al. developed an adaptive identification and control system based on online model maneuvering prediction for marine craft with an event-triggered mechanism [82]. In [83], Feng et al. developed an improved stabilization-diagram technique for real-time structural health monitoring (SHM) of civil structures based on the Eigensystem Realization Algorithm (ERA) and the Stochastic Subspace Identification (SSI) approach.

The large body of references found in the literature concerning the use of applied system identification methods for civil constructions and mechanical systems, in general, demonstrates the relevance of this significant topic, especially in the field of flexible engineering structures. However, this study is part of a wider research plan devised by the authors for investigating time-domain input–output applied system identification methods [84,85]. To this end, the system identification numerical procedures considered herein are the ARX, SSEST, N4SID, ERA/OKID, and TFEST methods [86,87,88,89,90], which are analyzed in detail below.

### 1.3. Scope and Contributions of This Investigation

This paper is devoted to numerically and experimentally analyzing the most relevant system identification methods found in the literature, which are based on input–output measurement sets recorded in the time domain, considering both the ease of implementation of the algorithms and their performance using numerical and experimental data. Therefore, the goal of this work is to perform a systematic review of this family of applied system identification techniques and subsequently to carry out a data-driven comparison of their performance based on a simple case study that is easily reproducible by other researchers. Furthermore, as the principal novelty and main contribution of this work, this investigation focuses on demonstrating and solving all the challenges concerning the practical implementation of all the system identification procedures mentioned in the paper, as well as in the elaboration of their numerical results and deduction from the experimental outcomes of peculiar dynamical features and reasonable inferences about physical quantities of engineering interest for the modal parameter analysis of structural systems. Thus, for the problem at hand, a simple vibrating structure is assumed as the demonstrative example in the paper. In contrast, the issue of experimental modal parameter identification is addressed and solved in the manuscript.

**Figure 1:**
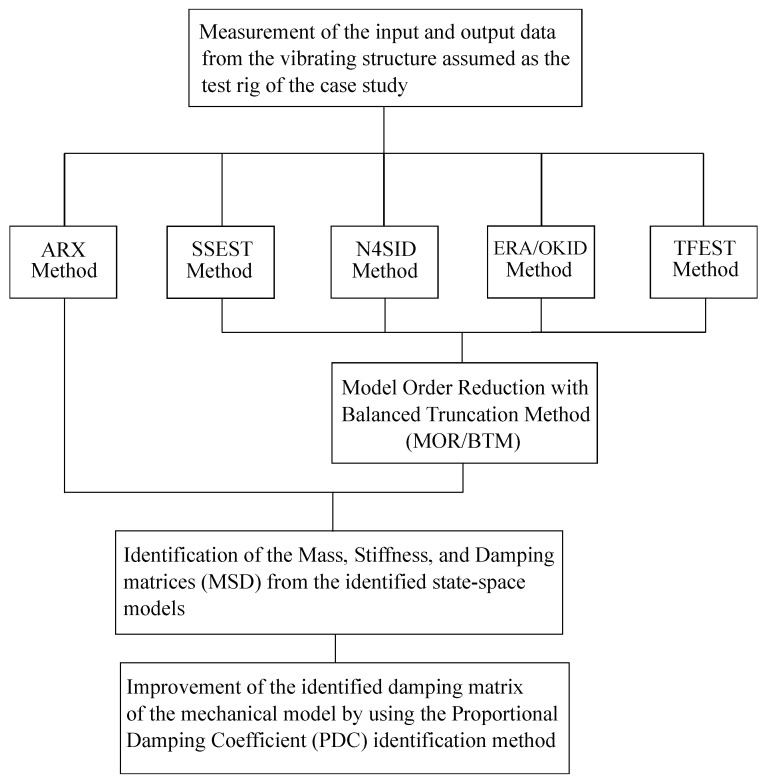
Conceptual flowchart of the proposed approach adopted in this study for performing a comparative analytical review followed by a numerical and experimental system identification analysis of the five system identification techniques considered in the paper.

To summarize the scope and the contributions of the work performed, as well as the approach followed in this investigation, a synthetic flowchart is reported in Figure 1.

As shown in Figure 1, the computational procedures considered in this paper for performing the applied system identification of dynamical models of mechanical systems are the ARX, SSEST, N4SID, ERA/OKID, and TFEST methods. The acronym ARX stands for Autoregressive Model with Exogenous Inputs [86,91]. The acronym SSEST stands for state-space estimation method [87,92]. The acronym N4SID stands for Numerical Algorithms for Subspace State-Space System Identification [2,88]. The acronym ERA/OKID stands for Eigensystem Realization Algorithm with Observer/Kalman Filter Identification method [1,89]. The acronym TFEST stands for transfer function estimation method [90,93]. Thus, a traditional identification method such as the ARX method, subspace identification approaches such as the SSEST and N4SID methods, and a combination of the eigensystem identification technique with the Kalman filter method such as the ERA/OKID method, and an identification method based on the transfer functions such as the TFEST method are employed in the paper for estimating a dynamical model of the vibrating structure assumed as the case study.

The engineering approach devised and proposed in this study consists of the four fundamental steps shown in Figure 1. The first step measures the numerical and experimental input and output signals of the studied dynamical system. In the second step, five different system identification methods, the ARX, SSEST, N4SID, ERA/OKID, and TFEST techniques, are utilized to estimate the system dynamic model. In particular, except for the ARX approach, the order of the identified model found using all the system identification methods examined in the paper is reduced with the model order reduction approach combined with the balanced truncation method (MOR/BTM) procedure. In the third step, the mass, stiffness, and damping (MSD) identification method is employed to estimate the mass, stiffness, and damping matrices of the vibrating structure by using the state-space models of the dynamical system identified before. However, the damping matrix of the MSD method is highly sensitive to the system noise. As a result, in the fourth and last step, the damping matrix of the two-story frame assumed as the case study is improved using the proportional damping coefficient (PDC) estimation method, representing the final step of the proposed approach.

This paper is part of a broader research plan devised by the authors devoted to investigating time-domain applied system identification methods for constructing first-order state-space models and second-order configuration-space models of mechanical systems, such as composite structures, lightweight mechanisms, robotic manipulators, rotating machines, and biomechanical systems [84,85,94]. In this vein, the ARX, SSEST, N4SID, ERA/OKID, and TFEST methods represent the system identification numerical procedures chosen for this purpose. The paper focuses first on revising the algorithmic aspects of the numerical procedures mentioned before, also including the description of the MOR/BTM method for obtaining first-order dynamical models of reduced dimensions, the MSD method for constructing second-order mechanical models from a first-order state-space realization, and the PDC method for improving the estimation of the identified damping matrix. Additionally, this paper proposes the results of numerical and actual experiments using a simple test rig. The case study considered in this paper is a typical example of a flexible structure of the shear type that can be mathematically modeled considering different degrees of complexity. Therefore, this structural system serves as a suitable example to test the efficiency and effectiveness of the time-domain system identification numerical procedures considered in this paper, mainly when applied to lightweight structures and flexible mechanisms.

In conclusion, the main contributions of this work lie in the comparative analysis carried out in this investigation and the combination of the eight methodological approaches mentioned above. The system identification methods considered herein are time-domain, input–output computational algorithms applicable to all types of flexible structures. This means that the main limitation of the proposed research approach is the use of time history testing signals of both the external excitations and the corresponding system response, such as vibration measurements. Thus, the outcome of this study leads to an improvement in the overall system identification process and paves the way for developing new algorithms based on the same spirit. This new family of time-domain identification algorithms could potentially construct reduced first-order and second-order dynamical models in a seamless framework with better estimates of dissipative effects.

### 1.4. Organization of the Manuscript

Apart from the current Introduction section, this work is organized as follows. Section 2 thoroughly describes the fundamental steps of the time-domain system identification procedures of interest for this work. Section 3 contains a detailed description of the vibrating system used as the demonstrative example of the paper. In Section 4, the core part of the manuscript is provided, thereby analyzing and discussing the numerical and experimental results found in this investigation and comparing the performance of all the system identification algorithms examined in the paper. Section 5 is the conclusive section of the paper, in which the summary of the work performed is provided together with the authors’ comments on the quality of the results found in the proposed comparative analysis, as well as some suggestions and recommendations for future research developments.

## 2. Time-Domain System Identification Methods

### 2.1. Mathematical Background

**Figure 2:**
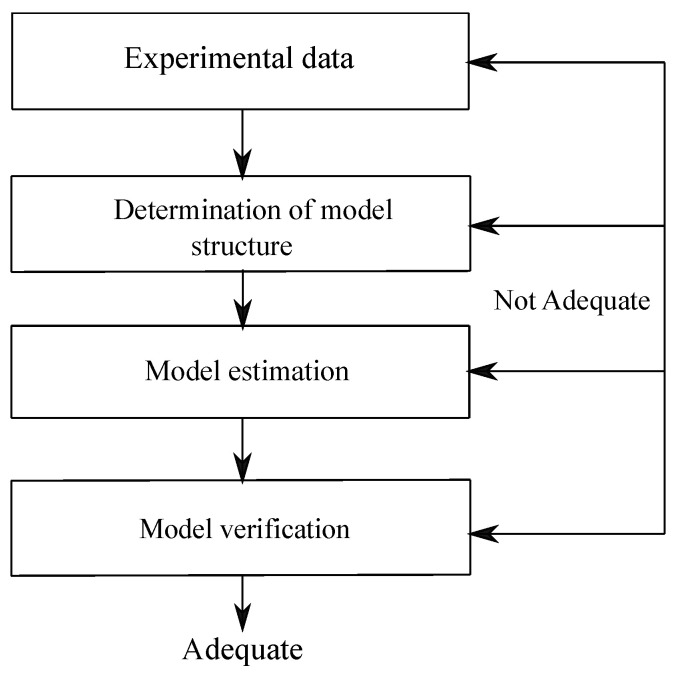
Conceptual flowchart of the logical steps followed in the system identification numerical procedures analyzed in the paper.

In this section, the key points of the time-domain system identification approaches of interest for this paper are illustrated in detail. These are the autoregressive exogenous (ARX) method, the state-space estimation (SSEST) method, the Numerical Algorithms for Subspace State-Space System Identification (N4SID), the Eigensystem Realization Algorithm (ERA) combined with the Observer/Kalman Filter Identification Methods (OKID), and the transfer function estimation method (TFEST). Subsequently, two methods for extracting second-order mechanical models from the identified first-order state-space dynamical models are discussed. These are the mass, stiffness, and damping (MSD) matrix identification method and the proportional damping coefficient (PDC) identification method. Finally, model order reduction with the balanced truncation method (MOR/BTM) for obtaining reduced mechanical models from identified input–output relationships is recalled. Figure 2 shows a schematic diagram that illustrates the fundamental steps and computational processes common to all the system identification algorithms analyzed in this work.

### 2.2. ARX Method

The acronym ARX stands for autoregressive model with exogenous inputs [86,91]. The use of the ARX model represents a traditional identification approach that is widely employed in the literature. In general, an ARX model has an autoregressive part, denoted with A(q)y(t), and an exogenous part, denoted with B(q)u(t), which can be represented in explicit form as follows:

(1) $$y\left(t\right)=\frac{B(q)}{A(q)}u\left(t\right)+\frac{1}{A(q)}w\left(t\right)$$

where *t* is the continuous-time variable, whereas u(t), y(t), and w(t), respectively, define the input vector with dimensions $n_{u}\times 1$, the output vector with dimensions $n_{y}\times 1$, and the white noise error term with dimensions $n_{e}\times 1$, with $n_{u}=n_{y}=n_{e}=N$. On the other hand, A(q) and B(q) are two different polynomials, expressed in terms of the variable *q* and constructed with unknown real coefficients, which can be defined as follows:

(2) $$A\left(q\right)=\sum_{i=1}^{n_{a}}a_{i}q^{-i}=1+a_{1}q^{-1}+a_{2}q^{-2}+\ldots +a_{n_{a}}q^{-n_{a}}$$

and

(3) $$B\left(q\right)=\sum_{j=1}^{n_{b}}b_{j}q^{-j}=b_{1}q^{-1}+b_{2}q^{-2}+\ldots +b_{n_{b}}q^{-n_{b}}$$

where $a_{i},i=1,2,\ldots ,n_{a}$ and $b_{j},j=1,2,\ldots ,n_{b}$ are the real coefficients associated with each term of the ARX polynomials denoted with A(q) and B(q), while $n_{a}$ and $n_{b}$ are their orders, respectively. The unknown coefficients of the polynomials that appear in the ARX model can be grouped into the following parameter vector:

(4) $$\vartheta ={\left[a_{1},a_{2},\ldots ,a_{n_{a}},b_{1},b_{2},\ldots ,b_{n_{b}}\right]}^{T}$$

where ϑ represents a vector of unknown parameters containing the real coefficients of the polynomials A(q) and B(q) having dimensions $\left(n_{a}+n_{b}\right)\times 1$. The set of unknown coefficients of the ARX polynomials can be readily estimated with an ordinary least-squares estimation method. To this end, one needs first to assemble the following coefficient matrix formed by the input and output measurements actually available for the dynamical system to be identified:

(5) $$\Phi =\left[\begin{array}{cc} Y & U \end{array}\right]$$

where

(6) $$Y=\left[\begin{array}{cccc} -y(n_{a}-1) & -y(n_{a}-2) & \ldots & -y(0)\\ -y(n_{a}) & -y(n_{a}-1) & \ldots & -y(1)\\ -y(n_{a}+1) & -y(n_{a}) & \ldots & -y(2)\\ \vdots & \vdots & \ddots & \vdots \\ -y(N-1) & -y(N-2) & \ldots & -y(N-n_{a}) \end{array}\right]$$

and

(7) $$U=\left[\begin{array}{cccc} u(n_{a}-1) & u(n_{a}-2) & \ldots & u(n_{a}-n_{b})\\ u(n_{a}) & u(n_{a}-1) & \ldots & u(n_{a}-n_{b}+1)\\ u(n_{a}+1) & u(n_{a}) & \ldots & u(n_{a}-n_{b}+2)\\ \vdots & \vdots & \ddots & \vdots \\ u(N-1) & u(N-2) & \ldots & u(N-n_{b}) \end{array}\right]$$

where it is assumed that $n_{u}=n_{y}=N$, as mentioned before, and Φ represents the regressor matrix, having dimensions $n_{y}\times \left(n_{a}+n_{b}\right)$, while Y and U are two submatrices of dimensions $n_{y}\times n_{a}$ and $n_{u}\times n_{b}$ that form the main regressor matrix Φ containing output and input measurements, respectively. Therefore, the least-squares estimation process arises from the rearrangement of Equation (1) in matrix form and leads to the following result:

(8) $$\hat{\vartheta }=\Phi^{+}y={\left(\Phi^{T}\Phi \right)}^{-1}\Phi y$$

where $\hat{\vartheta }$ represents an estimation of the unknown parameter vector of dimensions $\left(n_{a}+n_{b}\right)\times 1$ and $\Phi^{+}$ is the Moore–Penrose pseudo-inverse matrix of dimensions $\left(n_{a}+n_{b}\right)\times n_{y}$ associated with the regressor matrix Φ.

### 2.3. SSEST Method

The acronym SSEST stands for the state-space estimation method [87,92]. In this subsection, the main features of the SSEST method are described. The SSEST method is used to obtain the state-space model of a dynamic system using time-domain data or frequency-domain data. The SSEST method is based on initializing the model parameters using a subspace approach, such as the one based on the numerical methods for subspace state-space system identification, followed by the use of an iterative method that employs a prediction error and a minimization algorithm to improve the quality of the identified model. As discussed below, the initialization of the SSEST method is based on the use of the N4SID method. Therefore, the MOESP, CVA, and SSARX weighting matrices are analyzed when using the SSEST method, and the best result is typically obtained considering the CVA set of weighting matrices. On the other hand, the improvement in the estimation of the continuous-time state-space matrices, indicated as A, B, C, and D, with the use of the iterative method based on the SSEST technique is synthetically explained below.

The discretization of a continuous-time state-space model of a dynamical system leads to an approximate discrete-time state-space model given as follows:

(9) $$\left\{\begin{array}{c} z\left(k+1\right)=A_{d}z\left(k\right)+B_{d}u\left(k\right)\\ y(k)=\mathrm{Cz}(k)+\mathrm{Du}(k)+e(k) \end{array}\right.$$

where u(k), y(k), z(k), and e(k), respectively, represent the input vector, having dimensions $n_{u}\times 1$; the output vector, having dimensions $n_{y}\times 1$; the state vector, having dimensions $n_{z}\times 1$; and the error vector, having dimensions $n_{y}\times 1$; while $A_{d}$, $B_{d}$, C, and D denote the discrete-time matrices of the state-space model, having dimensions $n_{z}\times n_{z}$, $n_{z}\times n_{u}$, $n_{y}\times n_{z}$, and $n_{y}\times n_{u}$, respectively. The use of the maximum likelihood estimate (MLE) of the discrete-time matrices forming the state-space model leads to the following set of equations:

(10) min(e),e=e(y,z,u,C,D)

where

(11) $$e=\sum_{i=1}^{N}{∥y\left(k\right)-C\hat{z}\left(k\right)-\mathrm{Du}\left(k\right)∥}^{2}$$

and

(12) $$\hat{z}\left(k+1\right)=A_{d}\hat{z}\left(k\right)+B_{d}u\left(k\right)$$

where *e* represents the prediction error and the vector $\hat{z}\left(k\right)$ represents the estimation of the vector z(k). Considering the MLE approach, Equation (10), which represents the fundamental equation for the definition of the SSEST method, can be mathematically reformulated as follows:

(13) $$\min\left(V\right),\;V=V\left(y,u,A_{d},B_{d},C,D\right)$$

where

(14) $$V=\sum_{i=1}^{N}{∥y\left(k\right)-\hat{y}\left(k|A_{d},B_{d},C,D\right)∥}^{2}$$

and

(15) $$\hat{y}\left(k|A_{d},B_{d},C,D\right)=\mathrm{Du}\left(k\right)+C\sum_{j=1}^{k}\left(A_{d}^{k-j}B_{d}u\left(j\right)\right)$$

where *V* represents the performance index of the identification algorithm to be minimized and $\hat{y}\left(k\right)$ is the identified output vector associated with the vector y(k). It is worth noting that, for a fixed couple of the $A_{d}$ and C matrices, the vector $\hat{y}\left(k\right)$ is a linear function of the matrices $B_{d}$ and D, and, for a fixed couple of the $A_{d}$ and $B_{d}$ matrices, the vector $\hat{y}\left(k\right)$ is a linear function of the matrices C and D. Therefore, the matrices $B_{d}$ and D can be re-estimated through a simple linear least-squares estimation method for given iterative values of the matrices $A_{d}$ and C. Then, the matrices C and D can be re-estimated again through a least-squares estimation method for given iterative values of the matrices $A_{d}$ and $B_{d}$. After the matrices $B_{d}$, C, and D are re-estimated, the matrix $A_{d}$ can be re-estimated using Equation (13), and the re-estimated $B_{d}$, C, and D matrices can be obtained as well. The iterative identification process that characterizes the SSEST method ends when a prescribed tolerance for the error given by Equation (10) is met or when an assigned maximum number of finite iterations is reached.

### 2.4. N4SID Method

The acronym N4SID stands for Numerical Algorithms for Subspace State-Space System Identification method [2,88]. In this subsection, the fundamental steps of the N4SID method are described in detail. To this end, consider the following discrete-time state-space model of a general structural system:

(16) $$\left\{\begin{array}{c} \begin{array}{c} z\left(k+1\right)=A_{d}z\left(k\right)+B_{d}u\left(k\right)\\ y(k)=\mathrm{Cz}(k)+\mathrm{Du}(k) \end{array} \end{array}\right.$$

where *k* denotes the discrete time variable; z(k), y(k), and u(k), respectively, represent the discrete-time state vector with dimensions $n_{z}\times 1$, the discrete-time output vector with dimensions $n_{y}\times 1$, and the discrete-time input vector with dimensions $n_{u}\times 1$; while the matrices $A_{d}$, $B_{d}$, C, and D denote the discrete-time state matrix, having dimensions $n_{z}\times n_{z}$, the discrete-time input influence matrix, having dimensions $n_{z}\times n_{u}$, the output influence matrix, having dimensions $n_{y}\times n_{z}$, and the direct transmission matrix, having dimensions $n_{y}\times n_{u}$, respectively.

The discrete-time state-space model of a generic mechanical system can be rewritten with the use of the past and future inputs and outputs as follows:

(17) $$\begin{array}{c} \left\{\begin{array}{c} Y_{p}=\Gamma_{i}Z_{p}+H_{i}U_{p}\\ Y_{f}=\Gamma_{i}Z_{f}+H_{i}U_{f}\\ Z_{f}=A_{d}^{i}Z_{p}+\Delta_{i}U_{p} \end{array}\right. \end{array}$$

where *i* and *j* are two manipulable integer numbers. The matrices denoted with $\Gamma_{i}$, $\Delta_{i}$, and $H_{i}$ represent the observability matrix of dimensions $n_{y}i\times n_{z}$, the controllability matrix of dimensions $n_{z}\times n_{u}i$, and the discrete-time state-space Toeplitz matrix of dimensions $n_{y}i\times n_{u}i$, respectively. The matrices denoted with $U_{p}$, $U_{f}$, $Y_{p}$, and $Y_{f}$ are rectangular matrices that identify the Hankel matrix of the past inputs with dimensions $in_{u}\times j$, the Hankel matrix of future inputs with dimensions $in_{u}\times j$, the Hankel matrix of past outputs with dimensions $in_{y}\times j$, and the Hankel matrix of future outputs with dimensions $in_{y}\times j$, respectively. The matrices $U_{p}$, $U_{f}$, $Y_{p}$, and $Y_{f}$ can be, respectively, written as follows:

(18) $$U_{p}=\left[\begin{array}{cccc} u(0) & u(1) & \cdots & u(j-1)\\ u(1) & u(2) & \cdots & u(j)\\ \vdots & \vdots & \ddots & \vdots \\ u(i-1) & u(i) & \cdots & u(i+j-2) \end{array}\right]$$

(19) $$U_{f}=\left[\begin{array}{cccc} u(i) & u(i+1) & \cdots & u(i+j-1)\\ u(i+1) & u(i+2) & \cdots & u(i+j)\\ \vdots & \vdots & \ddots & \vdots \\ u(2i-1) & u(2i) & \cdots & u(2i+j-2) \end{array}\right]$$

(20) $$Y_{p}=\left[\begin{array}{cccc} y(0) & y(1) & \cdots & y(j-1)\\ y(1) & y(2) & \cdots & y(j)\\ \vdots & \vdots & \ddots & \vdots \\ y(i-1) & y(i) & \cdots & y(i+j-2) \end{array}\right]$$

(21) $$Y_{f}=\left[\begin{array}{cccc} y(i) & y(i+1) & \cdots & y(i+j-1)\\ y(i+1) & y(i+2) & \cdots & y(i+j)\\ \vdots & \vdots & \ddots & \vdots \\ y(2i-1) & y(2i) & \cdots & y(2i+j-2) \end{array}\right]$$

Theoretically, the parameters *i* and *j* can be chosen arbitrarily. However, as the length of the data record *l* and the dimension of the parameters *i* and *j* increase, the prediction success of the discrete-time state-space model increases. The past state matrix, denoted with $Z_{p}$ and having dimensions $n_{z}\times j$, and the future state matrix, denoted with $Z_{f}$ and having dimensions $n_{z}\times j$, can be written as follows:

(22) $$Z_{p}=\left[\begin{array}{cccc} z(0) & z(2) & \ldots & z(j-1) \end{array}\right]$$

and

(23) $$Z_{f}=\left[\begin{array}{cccc} z(i) & z(i+1) & \ldots & z(i+j-1) \end{array}\right]$$

The observability matrix, denoted by $\Gamma_{i}$, and the controllability matrix, denoted by $\Delta_{i}$, are explicitly given by the following matrix equations:

(24) $$\Gamma_{i}=\left[\begin{array}{c} C\\ CA_{d}\\ {\mathrm{CA}}_{d}^{2}\\ \vdots \\ {\mathrm{CA}}_{d}^{i-1} \end{array}\right]$$

and

(25) $$\Delta_{i}=\left[\begin{array}{ccccc} {A_{d}^{i-1}B}_{d} & {A_{d}^{i-2}B}_{d} & \cdots & A_{d}B_{d} & B_{d} \end{array}\right]$$

The triangular Toeplitz matrix $H_{i}$ can be assembled as follows:

(26) $$H_{i}=\left[\begin{array}{ccccc} D & O & O & \ldots & O\\ CB_{d} & D & O & \ldots & O\\ CA_{d}B_{d} & CB_{d} & D & \ldots & O\\ \vdots & \vdots & \vdots & \ddots & \vdots \\ {\mathrm{CA}}_{d}^{i-2}B_{d} & {\mathrm{CA}}_{d}^{i-3}B_{d} & {\mathrm{CA}}_{d}^{i-4}B_{d} & \ldots & D \end{array}\right]$$

where **O** represents a zero matrix with proper dimensions. The matrix of future states $Z_{f}$ can be rewritten with the matrix of past inputs, denoted by $U_{p}$, and the matrix of past outputs, denoted by $Y_{p}$. For this purpose, one can write the following:

(27) $$\begin{array}{cc} Z_{f} & =A_{d}^{i}Z_{p}+\Delta_{i}U_{p}\\ & =A_{d}^{i}\left(\Gamma_{i}^{+}Y_{p}-\Gamma_{i}^{+}H_{i}U_{p}\right)+\Delta_{i}U_{p}\\ & =\left(\Delta_{i}-A_{d}^{i}\Gamma_{i}^{+}H_{i}\right)U_{p}+A_{d}^{i}\Gamma_{i}^{+}Y_{p}\\ & =L_{p}W_{p} \end{array}$$

where the matrix $\Gamma_{i}^{+}$ represents the Moore–Penrose pseudo-inverse matrix of the matrix $\Gamma_{i}$. The matrix $L_{p}$, having dimensions $n_{z}\times i\left(n_{u}+n_{y}\right)$, and the matrix $W_{p}$, having dimensions $i(n_{u}+n_{y})\times j$, are, respectively, defined as follows:

(28) $$L_{p}=\left[\begin{array}{cc} \Delta_{i}-A_{d}^{i}\Gamma_{i}^{+}H_{i} & A_{d}^{i}\Gamma_{i}^{+} \end{array}\right],\;W_{p}=\left[\begin{array}{c} U_{p}\\ Y_{p} \end{array}\right]$$

The matrix of future outputs $Y_{f}$ can be rewritten with an organized version of the future state matrix $Z_{f}$ as follows:

(29) $$Y_{f}=\Gamma_{i}Z_{f}+H_{i}U_{f}=\Gamma_{i}L_{p}W_{p}+H_{i}U_{f}$$

Define the projection matrix onto the set of future inputs denoted by $\Pi_{U_{f}^{⊥}}$, having dimensions j×j, as

(30) $$\Pi_{U_{f}^{⊥}}=I-U_{f}^{T}{\left(U_{f}U_{f}^{T}\right)}^{+}U_{f}$$

By post-multiplying the matrix of future output $Y_{f}$ with the projection matrix onto the set of future inputs $\Pi_{U_{f}^{⊥}}$, the following two matrix equations are obtained:

(31) $$Y_{f}\Pi_{U_{f}^{⊥}}=\Gamma_{i}L_{p}W_{p}\Pi_{U_{f}^{⊥}},\;H_{i}U_{f}\Pi_{U_{f}^{⊥}}=O$$

For simplicity, define the following matrix of dimensions j×j denoted with ${\bar{W}}_{p}$ and given by

(32) $${\bar{W}}_{p}={\left(W_{p}\Pi_{U_{f}^{⊥}}\right)}^{+}W_{p}$$

The post-multiplication of Equation (31) by the matrix ${\bar{W}}_{p}$ can be written as follows:

(33) $$Y_{f}\Pi_{U_{f}^{⊥}}{\bar{W}}_{p}=\Gamma_{i}L_{p}W_{p}\Pi_{U_{f}^{⊥}}{\bar{W}}_{p}$$

Consequently, the following matrix equations can be deduced:

(34) $$O_{i}=Y_{f}\Pi_{U_{f}^{⊥}}{\bar{W}}_{p}=Y_{f}\Pi_{U_{f}^{⊥}}{\left(W_{p}\Pi_{U_{f}^{⊥}}\right)}^{+}W_{p}$$

and

(35) $$O_{i}=\Gamma_{i}L_{p}W_{p}=\Gamma_{i}Z_{f}$$

Finally, a fundamental matrix equation is obtained as follows:

(36) $$O_{i}=\Gamma_{i}Z_{f}$$

where the matrix $O_{i}$ is a rectangular matrix with dimensions $in_{y}\times j$. The fundamental matrix $O_{i}$ can be post-processed with the weighting matrices $W_{1}$ of dimensions $in_{y}\times j$ and $W_{2}$ of dimensions j×j as follows:

(37) $${\bar{O}}_{i}=W_{1}O_{i}W_{2}$$

where the matrix ${\bar{O}}_{i}$, with dimensions $in_{y}\times j$, defines the post-processed version of the fundamental matrix denoted by $O_{i}$. The specific definition of the weight matrices indicated as $W_{1}$ and $W_{2}$ significantly affects the success of the estimated model, as well as of the identification process when using the N4SID approach. There are various types of weighting matrices, such as the Multivariable Output-Error State Space (MOESP) method and the canonical variate analysis (CVA) method. In this paper, the weight matrices $W_{1}$ and $W_{2}$ are determined according to the CVA technique as follows:

(38) $$\left\{\begin{array}{c} W_{1}=E\left[\left(Y_{f}\Pi_{U_{f}^{⊥}}\right){\left(Y_{f}\Pi_{U_{f}^{⊥}}\right)}^{T}\right]{)}^{-1/2}\\ W_{2}=\Pi_{U_{f}^{⊥}} \end{array}\right.$$

where E[x] identifies the expected value of the variable *x*. The post-processed matrix ${\bar{O}}_{i}$ can be partitioned by a singular value decomposition (SVD) to yield

(39) $${\bar{O}}_{i}=U\Sigma V^{T}$$

where the matrix Σ with dimensions $in_{y}\times j$ consists of the singular value of the post-processed matrix indicated by ${\bar{O}}_{i}$, while the orthogonal matrix U with dimensions $in_{y}\times in_{y}$ and the orthogonal matrix V with dimensions j×j are square matrices obtained using singular value decomposition (SVD). As a result, the post-processed matrix ${\bar{O}}_{i}$ can be written as follows:

(40) $${\bar{O}}_{i}=U_{1}\Sigma_{1}V_{1}^{T}$$

where

(41) $$U=\left[\begin{array}{cc} U_{1} & U_{2} \end{array}\right],\;\Sigma =\left[\begin{array}{cc} \Sigma_{1} & O\\ O & O \end{array}\right],\;V^{T}=\left[\begin{array}{c} V_{1}^{T}\\ V_{2}^{T} \end{array}\right]$$

where the matrix $U_{1}$ with dimensions $in_{y}\times {\hat{n}}_{z}$, the matrix $U_{2}$ with dimensions $in_{y}\times \left(in_{y}-{\hat{n}}_{z}\right)$, the matrix $V_{1}$ with dimensions $j\times {\hat{n}}_{z}$, and the matrix $V_{2}$ with dimensions $j\times \left(j-{\hat{n}}_{z}\right)$ represent submatrices of U and V, which are obtained from the singular value decomposition of the post-processed matrix ${\bar{O}}_{i}$. Additionally, the matrix $\Sigma_{1}$, having dimensions ${\hat{n}}_{z}\times {\hat{n}}_{z}$, is a square diagonal matrix given as

(42) $$\Sigma_{1}=\mathrm{diag}\left(\sigma_{1},\sigma_{2},\ldots ,\sigma_{{\hat{n}}_{z}}\right)$$

where ${\hat{n}}_{z}$ is the number of nonzero singular values indicated with $\sigma_{h},\;h=1,2,\ldots ,{\hat{n}}_{z}$ and represents the dimensions of the estimated state-space model. At this stage, the fundamental matrix denoted by $O_{i}$ can be written with the weighting matrices as follows:

(43) $$O_{i}=W_{1}^{-1}U_{1}\Sigma_{1}V_{1}^{T}W_{2}^{-1}=\Gamma_{i}Z_{f}$$

The equation can be partitioned into two parts as follows:

(44) $$\left\{\begin{array}{c} \Gamma_{i}=W_{1}^{-1}U_{1}\Sigma_{1}^{1/2\;}T\\ Z_{f}=T^{-1}\Sigma_{1}^{1/2\;}V_{1}^{T}W_{2}^{-1} \end{array}\right.$$

where T denotes a similarity transformation matrix, having dimensions ${\hat{n}}_{z}\times {\hat{n}}_{z}$, which is an appropriate non-singular square matrix. The similarity transformation matrix T can also be assumed as the identity matrix to simplify the mathematical manipulations. By using these assumptions, the equations can be written as follows:

(45) $$\left\{\begin{array}{c} Z_{f}=\Sigma_{1}^{1/2\;}V_{1}^{T}W_{2}^{-1}\\ \Gamma_{i}=W_{1}^{-1}U_{1}\Sigma_{1}^{1/2\;} \end{array}\right.$$

The last step of the N4SID method is the extraction of discrete-time state-space matrices, denoted by $A_{d}$, $B_{d}$, C, and D, from Equation (45). Firstly, the discrete-time matrix $\hat{C}$, which is the identified output influence matrix, can be calculated with the first $n_{y}$ rows of the Toeplitz matrix $\Gamma_{i}$ as follows:

(46) $$\hat{C}=E_{n_{y}}^{T}\Gamma_{i}$$

where $E_{n_{y}}$ represents a proper Boolean matrix. The identified discrete-time state matrix, indicated with ${\hat{A}}_{d}$, can be calculated as

(47) $${\hat{A}}_{d}={\left(\underline{\Gamma_{i}}\right)}^{+}\bar{\Gamma_{i}}$$

where the matrix ${\left(\underline{\Gamma_{i}}\right)}^{+}$ represents the Moore–Penrose pseudo-inverse matrix of the matrix $\underline{\Gamma_{i}}$, while the matrix $\underline{\Gamma_{i}}$ with dimensions $\left(i-1\right)n_{y}\times n_{z}$ and the matrix $\bar{\Gamma_{i}}$ with dimensions $\left(i-1\right)n_{y}\times n_{z}$ represent the modified versions of the Toeplitz matrix $\Gamma_{i}$, in which the last matrix block and the first matrix block with dimensions $n_{y}\times n_{z}$ are removed, respectively. The modified versions of the Toeplitz matrix can be assembled as follows:

(48) $$\underline{\Gamma_{i}}=\left[\begin{array}{c} C\\ CA_{d}\\ {\mathrm{CA}}_{d}^{2}\\ \vdots \\ {\mathrm{CA}}_{d}^{i-2} \end{array}\right],\;\bar{\Gamma_{i}}=\left[\begin{array}{c} CA_{d}\\ {\mathrm{CA}}_{d}^{2}\\ {\mathrm{CA}}_{d}^{3}\\ \vdots \\ {\mathrm{CA}}_{d}^{i-1} \end{array}\right]$$

To estimate the identified discrete-time input influence matrix ${\hat{B}}_{d}$ and the identified direct transmission matrix $\hat{D}$, the matrix of the future output $Y_{f}$ is multiplied by the matrices $U_{f}^{+}$ and $\Gamma_{i}^{⊥}$ as follows:

(49) $$\Gamma_{i}^{⊥}Y_{f}U_{f}^{+}=\Gamma_{i}^{⊥}\Gamma_{i}Z_{f}U_{f}^{+}+\Gamma_{i}^{⊥}H_{i}U_{f}U_{f}^{+}=\Gamma_{i}^{⊥}H_{i}$$

where the matrix $U_{f}^{+}$ denotes the Moore–Penrose pseudo-inverse of the matrix of future inputs denoted with $U_{f}$, while $\Gamma_{i}^{⊥}$ represents the projection matrix associated with the observability matrix $\Gamma_{i}$ which is a full-rank matrix. To simplify the final equations, the following mathematical manipulations can be exploited:

(50) $$N=\Gamma_{i}^{⊥}Y_{f}U_{f}^{+},\;P=\Gamma_{i}^{⊥}$$

where the matrix N with dimensions $n_{z}\times in_{u}$ and the matrix P with dimensions $n_{z}\times in_{y}$ are appropriate rectangular matrices. By using the previous matrix factorizations and the simplifications given by Equation (50), Equation (49) can be rewritten as follows:

(51) $$N=PH_{i}$$

After that, Equation (51) can be expanded as follows:

(52) $$\left[\begin{array}{cccc} N_{1} & N_{2} & \ldots & N_{i} \end{array}\right]=\left[\begin{array}{cccc} P_{1} & P_{2} & \ldots & P_{i} \end{array}\right]H_{i}$$

Thus, Equation (52) can be rewritten as follows:

(53) $$\left[\begin{array}{c} N_{1}\\ N_{2}\\ \vdots \\ N_{i} \end{array}\right]=\left[\begin{array}{cccc} P_{1} & P_{2} & \ldots & P_{i}\\ P_{2} & P_{3} & \ldots & O\\ \vdots & \vdots & \ddots & \vdots \\ P_{i} & O & \ldots & O \end{array}\right]\left[\begin{array}{cc} I & O\\ O & \underline{\Gamma_{i}} \end{array}\right]\Omega$$

where the matrix Ω with dimensions $\left(n_{z}+n_{y}\right)\times n_{u}$ is a rectangular matrix consisting of the identified versions of the matrices $B_{d}$ and D. This matrix is defined as

(54) $$\Omega =\left[\begin{array}{c} D\\ B_{d} \end{array}\right]$$

Therefore, one obtains the following matrix equation:

(55) $$\Omega ={\left[\begin{array}{cc} I & O\\ O & \underline{\Gamma_{i}} \end{array}\right]}^{+}{\left[\begin{array}{cccc} P_{1} & P_{2} & \ldots & P_{i}\\ P_{2} & P_{3} & \ldots & O\\ \vdots & \vdots & \ddots & \vdots \\ P_{i} & O & \ldots & O \end{array}\right]}^{+}\left[\begin{array}{c} N_{1}\\ N_{2}\\ \vdots \\ N_{i} \end{array}\right]$$

where the plus superscript symbol indicates the Moore–Penrose pseudo-inverse matrix. Finally, the identified discrete-time input influence matrix ${\hat{B}}_{d}$ and the identified discrete-time direct transmission matrix $\hat{D}$ are determined as follows:

(56) $$\hat{D}=E_{n_{y}}^{T}\Omega ,\;{\hat{B}}_{d}=E_{n_{z}}^{T}\Omega$$

where $E_{n_{y}}$ is a proper Boolean matrix that is used to obtain the matrix $\hat{D}$ from the first $n_{y}$ rows of the matrix Ω, while $E_{n_{z}}$ is a proper Boolean matrix that is used to obtain the matrix ${\hat{B}}_{d}$ from the last $n_{z}$ rows of the matrix Ω. In synthesis, the identified discrete-time state-space matrices can be written using the N4SID method as follows:

(57) $$\left\{\begin{array}{c} {\hat{A}}_{d}={\left(\underline{\Gamma_{i}}\right)}^{+}\bar{\Gamma_{i}}\\ {\hat{B}}_{d}=E_{n_{z}}^{T}\Omega \\ \hat{C}=E_{n_{y}}^{T}\Gamma_{i}\\ \hat{D}=E_{n_{y}}^{T}\Omega \end{array}\right.$$

where ${\hat{n}}_{z}$ is the dimension of the identified state, ${\hat{n}}_{u}=n_{u}$ and ${\hat{n}}_{y}=n_{y}$, respectively, denote the dimensions of the input and output measurements, whereas ${\hat{A}}_{d}$, ${\hat{B}}_{d}$, $\hat{C}$, and $\hat{D}$, respectively, represent the identified discrete-time state matrix of dimensions ${\hat{n}}_{z}\times {\hat{n}}_{z}$, the identified discrete-time input influence matrix of dimensions ${\hat{n}}_{z}\times {\hat{n}}_{u}$, the identified output influence matrix of dimensions ${\hat{n}}_{y}\times {\hat{n}}_{u}$, and the identified direct transmission matrix of dimensions ${\hat{n}}_{y}\times {\hat{n}}_{u}$ obtained with the use of the N4SID method.

### 2.5. ERA/OKID Method

The acronym ERA/OKID stands for the Eigensystem Realization Algorithm combined with the Observer/Kalman Filter Identification method [1,89]. In this subsection, the main features of the ERA/OKID method are thoroughly explained. The identification approach based on the ERA/OKID algorithm represents a method that estimates the discrete-time state-space model of a mechanical system using the state Markov parameters, the observer Markov parameters, and the observer gain Markov parameters. These parameters can be numerically obtained from measured input–output data for the system of interest.

A discrete-time state-space model of a mechanical system can be written as follows:

(58) $$\left\{\begin{array}{c} \begin{array}{c} z\left(k+1\right)=A_{d}z\left(k\right)+B_{d}u\left(k\right)\\ y(k)=\mathrm{Cz}(k)+\mathrm{Du}(k) \end{array} \end{array}\right.$$

where *k* denotes the discrete time variable, z(k), y(k), and u(k), respectively, represent the discrete-time state vector with dimensions $n_{z}\times 1$, the discrete-time output vector with dimensions $n_{y}\times 1$, and the discrete-time input vector with dimensions $n_{u}\times 1$, while the matrices $A_{d}$, $B_{d}$, C, and D denote the discrete-time state matrix, having dimensions $n_{z}\times n_{z}$; the discrete-time input influence matrix, having dimensions $n_{z}\times n_{u}$; the output influence matrix, having dimensions $n_{y}\times n_{z}$; and the direct transmission matrix, having dimensions $n_{y}\times n_{u}$, respectively. An observer can be added to the system to increase the accuracy of the transient dynamic analysis. For this purpose, the following state-space model can be introduced:

(59) $$\left\{\begin{array}{c} \begin{array}{c} \hat{z}\left(k+1\right)={\bar{A}}_{d}\hat{z}\left(k\right)+{\bar{B}}_{d}v\left(k\right)\\ \hat{y}\left(k\right)=\bar{C}\hat{z}\left(k\right)+\bar{D}u\left(k\right) \end{array} \end{array}\right.$$

where $\hat{z}\left(k\right)$, $\hat{y}\left(k\right)$, and v(k), respectively, represent the estimated state vector with dimensions $n_{z}\times 1$, the estimated measurement vector with dimensions $n_{y}\times 1$, and the generalized input vector with dimensions $\left(n_{u}+n_{y}\right)\times 1$, while ${\bar{A}}_{d}$ represents the discrete-time observer state matrix with dimensions $n_{z}\times n_{z}$, and ${\bar{B}}_{d}$ represents the discrete-time observer state influence matrix with dimensions $n_{z}\times \left(n_{u}+n_{y}\right)$. The observer state matrix, denoted with ${\bar{A}}_{d}$, the observer state influence matrix, denoted with ${\bar{B}}_{d}$, and the generalized input vector, denoted by v(k), are determined as follows:

(60) $${\bar{A}}_{d}=A_{d}+\mathrm{GC},\;{\bar{B}}_{d}=\left[\begin{array}{cc} B_{d}+\mathrm{GD} & -G \end{array}\right]$$

and

(61) $$v\left(k\right)={\left[\begin{array}{cc} u^{T}\left(k\right) & y^{T}\left(k\right) \end{array}\right]}^{T}$$

where G denotes the observer matrix with dimensions $n_{z}\times n_{y}$. The purpose of the observer matrix G is to adjust the eigenvalues of the matrix to make the observer state matrix asymptotically stable. Considering the effect of the introduction of the observer matrix in the definition of the sets of Markov parameters, two different input–output relationships of the discrete-time state-space model and the discrete-time observer state-space model are, respectively, defined as follows:

(62) $$\left\{\begin{array}{c} y\left(k\right)=\sum_{j=0}^{k}\left(Y_{j}u\left(k-j\right)\right)\\ \hat{y}\left(k\right)=\sum_{j=0}^{k}\left({\bar{Y}}_{j}v\left(k-j\right)\right) \end{array}\right.$$

where $Y_{k}$ is a rectangular matrix with dimensions $n_{y}\times n_{u}$ representing discrete impulse response function defining the system Markov parameters, while ${\bar{Y}}_{k}$ represents the observer Markov parameters with dimensions $n_{y}\times \left(n_{u}+n_{y}\right)$. The system and observer Markov parameters are, respectively, determined as the following:

(63) $$\left\{\begin{array}{c} Y_{0}=D\\ Y_{k}={\mathrm{CA}}_{d}^{k-1}B_{d} \end{array},\;\right.\left\{\begin{array}{c} {\bar{Y}}_{0}=D\\ {\bar{Y}}_{k}={C\bar{A}}_{d}^{k-1}{\bar{B}}_{d} \end{array}\right.$$

The observer Markov parameters can be obtained from experimental input–output data using a simple least-squares approach. In this way, an input–output relationship of a discrete-time dynamical model can be written as follows:

(64) $$y\left(k\right)+\sum_{j=1}^{p}\left({\bar{Y}}_{j}^{\left(2\right)}y\left(k-j\right)\right)=\sum_{j=1}^{p}\left({\bar{Y}}_{j}^{\left(1\right)}u\left(k-j\right)\right)+\mathrm{Du}\left(k\right)$$

where the matrix ${\bar{Y}}^{\left(1\right)}$ with dimensions $n_{y}\times n_{u}$ and the matrix ${\bar{Y}}^{\left(2\right)}$ with dimensions $n_{y}\times n_{y}$ can be, respectively, written as

(65) $$\left\{\begin{array}{c} {\bar{Y}}_{k}^{\left(1\right)}=C{\left(A_{d}+\mathrm{GC}\right)}^{k-1}\left(B_{d}+\mathrm{GD}\right)\\ {\bar{Y}}_{k}^{\left(2\right)}=C{\left(A_{d}+\mathrm{GC}\right)}^{k-1}G \end{array}\right.$$

Additionally, the observer Markov parameters, denoted with ${\bar{Y}}_{k}$, can be written in a matrix form as follows:

(66) $${\bar{Y}}_{k}=\left[\begin{array}{cc} {\bar{Y}}_{k}^{\left(1\right)} & -{\bar{Y}}_{k}^{\left(2\right)} \end{array}\right]$$

An assembled matrix, denoted with $\Gamma_{k}$, consisting of the system Markov parameters $Y_{k}$ and the observer gain Markov parameters $\Gamma_{k}$, can be explicitly constructed as

(67) $$\Gamma_{k}=\left[\begin{array}{cc} Y_{k} & Y_{k}^{0} \end{array}\right]$$

where the matrix $\Gamma_{k}$, with dimensions $n_{y}\times \left(n_{u}+n_{y}\right)$, consists of an assembly of the fundamental sets of Markov parameters. The generalized block Hankel matrix can be rewritten with the use of the matrix $\Gamma_{k}$ as the following:

(68) $$H\left(k-1\right)=\left[\begin{array}{cccc} \Gamma_{k} & \Gamma_{k+1} & \cdots & \Gamma_{k+\gamma -1}\\ \Gamma_{k+1} & \Gamma_{k+2} & \cdots & \Gamma_{k+\gamma }\\ \vdots & \vdots & \ddots & \vdots \\ \Gamma_{k+p-1} & \Gamma_{k+p} & \cdots & \Gamma_{k+p+\gamma -2} \end{array}\right]$$

where the matrix denoted by H(k−1), with dimensions $pn_{y}\times \gamma \left(n_{u}+n_{y}\right)$, represents the generalized block Hankel matrix, whereas *p* and γ are proper integers. The Hankel matrix H(0) can be readily determined for k=1 as follows:

(69) $$H\left(0\right)=\left[\begin{array}{cccc} \Gamma_{1} & \Gamma_{2} & \cdots & \Gamma_{\gamma }\\ \Gamma_{2} & \Gamma_{3} & \cdots & \Gamma_{\gamma +1}\\ \vdots & \vdots & \ddots & \vdots \\ \Gamma_{p} & \Gamma_{p+1} & \cdots & \Gamma_{p+\gamma -1} \end{array}\right]=P_{p}Q_{\gamma }$$

where the matrix, having dimensions $pn_{y}\times n_{z}$, denoted by $P_{p}$ represents the observability matrix of the dynamical system, whereas the matrix $Q_{\gamma }$ with dimensions $n_{z}\times \gamma \left(n_{u}+n_{y}\right)$ represents the controllability matrix of the dynamical system. The mathematical structure of both the observability matrix $P_{p}$ and the controllability matrix $Q_{\gamma }$ can be further manipulated as follows:

(70) $$P_{p}=\left[\begin{array}{c} C\\ CA_{d}\\ {\mathrm{CA}}_{d}^{2}\\ \vdots \\ {\mathrm{CA}}_{d}^{p-1} \end{array}\right]$$

and

(71) $$Q_{\gamma }=\left[\begin{array}{ccccc} {\tilde{B}}_{d} & A_{d}{\tilde{B}}_{d} & A_{d}^{2}{\tilde{B}}_{d} & \cdots & A_{d}^{\gamma -1}{\tilde{B}}_{d} \end{array}\right]$$

where ${\tilde{B}}_{d}$ is a matrix with dimensions $n_{z}\times \left(n_{u}+n_{y}\right)$ that is constructed with the system discrete-time input influence matrix, denoted by $B_{d}$, and the observer matrix, denoted with G. This matrix is given by

(72) $${\tilde{B}}_{d}=\left[\begin{array}{cc} B_{d} & G \end{array}\right]$$

After the introduction of the system state-space model modified by the presence of the observer, the generalized Hankel matrix H(0) for k=1 can be partitioned with the singular value decomposition method as the following:

(73) $$H\left(0\right)=R\Sigma S^{T}$$

where R and S are square orthonormal matrices with dimensions $pn_{y}\times pn_{y}$ and $\gamma \left(n_{u}+n_{y}\right)\times \gamma \left(n_{u}+n_{y}\right)$, respectively, whereas Σ is a nonzero rectangular matrix with dimensions $pn_{y}\times \gamma \left(n_{u}+n_{y}\right)$, which is defined as follows:

(74) $$\Sigma =\left[\begin{array}{cc} \Sigma_{{\hat{n}}_{z}} & O\\ O & O \end{array}\right]$$

where O is a proper zero matrix, ${\hat{n}}_{z}$ is the number of the identified singular values corresponding to the dimensions of the identified state-space model, while the square diagonal matrix, denoted with $\Sigma_{{\hat{n}}_{z}}$ and having dimensions ${\hat{n}}_{z}\times {\hat{n}}_{z}$, is given by the following equation:

(75) $$\Sigma_{{\hat{n}}_{z}}=\mathrm{diag}\left(\sigma_{1},\sigma_{2},\ldots ,\sigma_{{\hat{n}}_{z}}\right)$$

where the identified singular values are denoted as $\sigma_{i},\;i=1,2,\ldots ,{\hat{n}}_{z}$. The structure of the generalized Hankel matrix H(0) can be mathematically manipulated as

(76) $$H\left(0\right)=R_{{\hat{n}}_{z}}\Sigma_{{\hat{n}}_{z}}S_{{\hat{n}}_{z}}^{T},\;H^{+}\left(0\right)=S_{{\hat{n}}_{z}}\Sigma_{{\hat{n}}_{z}}^{-1}R_{{\hat{n}}_{z}}^{T}$$

where the rectangular matrices denoted with $R_{{\hat{n}}_{z}}$ and $S_{{\hat{n}}_{z}}$, respectively, represent the first ${\hat{n}}_{z}$ columns of the matrices R and S, while the matrix $H^{+}\left(0\right)$ with dimensions $\gamma \left(n_{u}+n_{y}\right)\times pn_{y}$ indicates the Moore–Penrose pseudo-inverse matrix of the generalized Hankel matrix, denoted with H(0). The Hankel matrix H(0) can be rewritten to extract the observability matrix indicated by ${\hat{P}}_{p}$ and the controllability matrix indicated by ${\hat{Q}}_{\gamma }$ as the following:

(77) $$H\left(0\right)=\left(R_{{\hat{n}}_{z}}\Sigma_{{\hat{n}}_{z}}^{1/2}\right)\left(\Sigma_{{\hat{n}}_{z}}^{1/2}S_{{\hat{n}}_{z}}^{T}\right)={\hat{P}}_{p}{\hat{Q}}_{\gamma }$$

which leads to

(78) $$\left\{\begin{array}{c} {\hat{P}}_{p}=R_{{\hat{n}}_{z}}\Sigma_{{\hat{n}}_{z}}^{1/2}\\ {\hat{Q}}_{\gamma }=\Sigma_{{\hat{n}}_{z}}^{1/2}S_{{\hat{n}}_{z}}^{T} \end{array}\right.$$

where ${\hat{P}}_{p}$ and ${\hat{Q}}_{\gamma }$ refer to the identified versions of the observability and controllability matrices. By using Equation (78), the matrix ${\hat{B}}_{d}$ and the matrix $\hat{C}$ can be obtained by extracting the first $n_{u}$ columns from the identified controllability matrix ${\hat{Q}}_{\gamma }$ and by extracting the first $n_{y}$ rows of the identified observability matrix ${\hat{P}}_{p}$, respectively. Subsequently, the generalized Hankel matrix, denoted with H(1) for k=2, can be described to estimate the identified system state matrix ${\hat{A}}_{d}$ as the following:

(79) $$H\left(1\right)=\left[\begin{array}{cccc} \Gamma_{2} & \Gamma_{3} & \cdots & \Gamma_{\gamma +1}\\ \Gamma_{3} & \Gamma_{4} & \cdots & \Gamma_{\gamma +2}\\ \vdots & \vdots & \ddots & \vdots \\ \Gamma_{p+1} & \Gamma_{p+2} & \cdots & \Gamma_{p+\gamma } \end{array}\right]={\hat{P}}_{p}A_{d}{\hat{Q}}_{\gamma }$$

The identified versions of the discrete-time state-space matrices are estimated with the use of the sets of Markov parameters based on the measured input–output signals and the definition of the generalized Hankel matrix. To conclude, the principal formulas for obtaining the identified discrete-time state-space model can be summarized as follows:

(80) $$\left\{\begin{array}{c} {\hat{A}}_{d}=\Sigma_{{\hat{n}}_{z}}^{-1/2}R_{{\hat{n}}_{z}}^{T}H\left(1\right)S_{{\hat{n}}_{z}}\Sigma_{{\hat{n}}_{z}}^{-1/2}\\ \left[\begin{array}{cc} {\hat{B}}_{d} & \hat{G} \end{array}\right]=\Sigma_{{\hat{n}}_{z}}^{1/2}S_{{\hat{n}}_{z}}^{T}E_{n_{u}+n_{y}}\\ \hat{C}=E_{n_{y}}^{T}R_{{\hat{n}}_{z}}\Sigma_{{\hat{n}}_{z}}^{1/2}\\ \hat{D}=Y_{0} \end{array}\right.$$

where ${\hat{n}}_{z}$ denotes the dimension of the identified state, ${\hat{n}}_{u}=n_{u}$ and ${\hat{n}}_{y}=n_{y}$, respectively, represent the dimensions of the input and output measurements, whereas the matrices ${\hat{A}}_{d}$, ${\hat{B}}_{d}$, $\hat{C}$, $\hat{D}$, and $\hat{G}$, respectively, indicate the identified version of the discrete-time state matrix of dimensions ${\hat{n}}_{z}\times {\hat{n}}_{z}$, the identified version of the discrete-time input influence matrix of dimensions ${\hat{n}}_{z}\times {\hat{n}}_{u}$, the identified version of the output influence matrix of dimensions ${\hat{n}}_{y}\times {\hat{n}}_{z}$, the identified version of the direct transmission matrix of dimensions ${\hat{n}}_{y}\times {\hat{n}}_{u}$, and the identified version of the observer matrix of dimensions ${\hat{n}}_{z}\times {\hat{n}}_{y}$ obtained with the use of the ERA/OKID method, whereas $E_{n_{y}}$ and $E_{n_{u}+n_{y}}$ are proper Boolean matrices necessary for extracting the matrices of interest.

### 2.6. TFEST Method

In the literature, there are two basic approaches to the continuous-time identification method. These are the direct approach and the indirect approach. The indirect approach consists of two parts. Firstly, the model of the system is identified with the discrete-time identification methods. Subsequently, the identified discrete-time model is translated to the continuous-time model. In the direct approach, the mathematical model of the system is directly identified with the TFEST method [95,96].

A single-input single-output continuous-time linear system can be described as follows:

(81) y(t)=G(p)u(t)

where

(82) $$G\left(p\right)=\frac{B(p)}{A(p)}=\frac{b_{0}+b_{1}p+\ldots b_{m}p^{m}}{a_{0}+a_{1}p+\ldots a_{n}p^{n}},\;a_{0}=1$$

where *p* is the differential operator, the elements u(t) and y(t) are, respectively, input and output signals of the system, G(p) represents the identified model of the system, the coefficients *a* and *b* are, respectively, the numerator and denominator coefficients of the identified model, while *m* and *n* denote the order of the numerator and the order of the denominator, respectively.

The system characteristic equation can be written with the input and output values as follows:

(83) $$a_{0}y_{u}\left(t\right)+a_{1}y_{u}^{\left(1\right)}\left(t\right)+\ldots +y_{u}^{\left(n\right)}\left(t\right)=b_{0}u\left(t\right)+b_{1}u^{\left(1\right)}\left(t\right)+\ldots +b_{m}u^{\left(m\right)}\left(t\right)$$

When the Laplace transform is applied to both sides of the previous equation, the resulting equation can be written as

(84) $$A\left(s\right)Y_{u}\left(s\right)=B\left(s\right)U\left(s\right)+C\left(s\right)$$

where

(85) $$A\left(s\right)=\sum_{i=0}^{n-1}a_{i}s^{i}+s^{n},\;B\left(s\right)=\sum_{i=0}^{m}b_{i}s^{i},\;C\left(s\right)=\sum_{i=0}^{n-1}c_{i}s^{i}$$

where *s* denotes the Laplace variable, whereas $Y_{u}\left(s\right)$ and U(s), respectively, represent the Laplace transform of the output and input signals, which are, respectively, indicated with $y_{u}\left(t\right)$ and u(t). When the filter is applied to both sides of the previous equation, one obtains

(86) $$A\left(s\right)F\left(s\right)Y_{u}\left(s\right)=B\left(s\right)F\left(s\right)U\left(s\right)+F\left(s\right)C\left(s\right)$$

The TFEST method can, therefore, estimate the continuous-time transfer function of a dynamical system with multiple poles by using all the input and output signals provided to the algorithm for both single-input single-output (SISO) and multiple-input multiple-output (MIMO) systems.

### 2.7. MSD Method

The acronym MSD stands for identification of the mass, stiffness, and damping matrices [66,68]. This subsection describes the key points of the MSD method used to obtain the configuration-space matrices of a mechanical system using an identified continuous-time state-space model of the same dynamical system. Sometimes, this method is also referred to as an inverse vibration problem since it finds the mass, stiffness, and damping matrices of a given structural system using the measured input and output datasets. The most important requirement of the MSD method is that there must be at least one co-located actuator–sensor pair for each degree of freedom of the mechanical system under study. To this end, an alternative representation of the continuous-time state-space model of a linear dynamic system can be written as follows:

(87) $$\left\{\begin{array}{c} R\dot{x}\left(t\right)+M\ddot{x}\left(t\right)=-\mathrm{Kx}\left(t\right)+B_{f}u\left(t\right)\\ M\dot{x}\left(t\right)=M\dot{x}\left(t\right) \end{array}\right.$$

where M, K, and R represent the system mass matrix, having dimensions $n_{x}\times n_{x}$; the system stiffness matrix, having dimensions $n_{x}\times n_{x}$; and the system damping matrix, having dimensions $n_{x}\times n_{x}$, respectively. Thus, one can rewrite the state-space representation of the system equations of motion in a compact matrix form as follows:

(88) $$U\dot{z}\left(t\right)=\mathrm{Vz}\left(t\right)+\mathrm{Eu}\left(t\right)$$

where U and V are square symmetric matrices with dimensions $n_{z}\times n_{z}$, whereas E is a rectangular matrix with dimensions $n_{z}\times n_{u}$. The set of state-space matrices given by U, V, and E represent the system transition matrices. The system transition matrices can be explicitly written as the following:

(89) $$U=\left[\begin{array}{cc} R & M\\ M & O \end{array}\right],\;V=\left[\begin{array}{cc} -K & O\\ O & M \end{array}\right],\;E=\left[\begin{array}{c} B_{f}\\ O \end{array}\right]$$

where O represents a zero matrix with appropriate dimensions. Since the associated eigenvalue problem of the alternative continuous-time state-space model is symmetric, this problem can be written as the following:

(90) VΨ=UΨΛ

where Λ and Ψ are square matrices with dimensions $n_{z}\times n_{z}$. In particular, Λ is a diagonal matrix containing the eigenvalues and Ψ is a full matrix containing the eigenvectors of the continuous-time state-space model. The eigenvector matrix denoted by Ψ can be separated into two parts as follows:

(91) $$\Psi =\left[\begin{array}{c} W\\ W\Lambda \end{array}\right]$$

where the rectangular matrix W, having dimensions $n_{x}\times n_{z}$, represents the eigenvector relative to the system configuration-space physical coordinates, while the square matrix Ψ, having dimensions $n_{z}\times n_{z}$, identifies the eigenvector associated with the system state-space mathematical coordinates. The eigenvector matrix denoted by W is directly associated with the eigenvector matrix denoted by Φ as follows:

(92) $$W=\left[\begin{array}{ccccccc} \varphi_{1} & \varphi_{1}^{*} & \varphi_{2} & \varphi_{2}^{*} & \ldots & \varphi_{n_{x}} & \varphi_{n_{x}}^{*} \end{array}\right]$$

and

(93) $$\Phi =\left[\begin{array}{cccc} \varphi_{1} & \varphi_{2} & \ldots & \varphi_{n_{x}} \end{array}\right]$$

where $\varphi_{j}$ is a generic vector with dimensions $n_{x}\times 1$ that represents the eigenvector of the second-order configuration-space model, and $\varphi_{j}^{*}$ represents the corresponding complex conjugate of the generic vector $\varphi_{j}$, with $j=1,2,\ldots ,n_{x}$. When all modes of the dynamic model of the mechanical system are underdamped, the system continuous-time eigenvalue matrix indicated by Λ can be written as

(94) $$\Lambda =\mathrm{diag}(\lambda_{1},\lambda_{2},\ldots ,\lambda_{n_{z}-1},\lambda_{n_{z}})$$

where

(95) $$\lambda_{j}=a_{j}\pm ib_{j}=-\xi_{j}\omega_{n,j}\pm i\sqrt{1-\xi_{j}^{2}}\omega_{n,j},\;j=1,2,\ldots ,n_{x}$$

where $i=\sqrt{-1}$ represents the imaginary unit, whereas $\lambda_{j}$, $a_{j}$, and $b_{j}$ represent the continuous-time state-space eigenvalue, the real part, and the imaginary part of the eigenvalue labeled with the integer $j=1,2,\ldots ,n_{x}$, respectively. Additionally, $\omega_{n,j}$ and $\xi_{j}$, respectively, identify the natural angular frequency and the dimensionless damping ratio of the generic mode shape $j=1,2,\ldots ,n_{x}$. At this stage, assume the following scaling of the state-space transition matrices:

(96) $$\Psi^{T}U\Psi =I\;\Leftrightarrow \;{\left[\begin{array}{c} W\\ W\Lambda \end{array}\right]}^{T}\left[\begin{array}{cc} R & M\\ M & O \end{array}\right]\left[\begin{array}{c} W\\ W\Lambda \end{array}\right]=I$$

and

(97) $$\Psi^{T}V\Psi =\Lambda \;\Leftrightarrow \;{\left[\begin{array}{c} W\\ W\Lambda \end{array}\right]}^{T}\left[\begin{array}{cc} -K & O\\ O & M \end{array}\right]\left[\begin{array}{c} W\\ W\Lambda \end{array}\right]=\Lambda$$

where I represents an identity matrix with appropriate dimensions. Equations (96) and (97) can be mathematically manipulated as follows:

(98) $$W^{T}\mathrm{RW}+W^{T}\mathrm{MW}\Lambda +\Lambda^{T}W^{T}\mathrm{MW}=I$$

and

(99) $$-W^{T}\mathrm{KW}+\Lambda^{T}W^{T}\mathrm{MW}\Lambda =\Lambda$$

By using the assumptions leading to these equations, the modal representation of the continuous-time state-space model can be written as follows:

(100) $$\left\{\begin{array}{c} {\dot{z}}_{m}\left(t\right)=A_{m}z_{m}\left(t\right)+B_{m}u\left(t\right)\\ y\left(t\right)=C_{m}z_{m}\left(t\right)+D_{m}u\left(t\right) \end{array}\right.$$

where $z_{m}$ represents the modal state vector with dimensions $n_{z}\times 1$, $A_{m}$ denotes the continuous-time modal state matrix with dimensions $n_{z}\times n_{z}$, $B_{m}$ represents the continuous-time modal input influence matrix with dimensions $n_{z}\times n_{u}$, and $C_{m}$ represents the modal output influence matrix with dimensions $n_{y}\times n_{z}$, whereas $D_{m}$ denotes the modal direct transmission matrix with dimensions $n_{y}\times n_{u}$. The continuous-time state-space modal matrices can be written as follows:

(101) $$A_{m}=\Lambda ,\;B_{m}=W^{T}B_{f},\;C_{m}=C_{s}W\Lambda^{b},\;D_{m}=D$$

where $C_{s}$ is a rectangular matrix, having dimensions $n_{y}\times n_{z}$, associated with the type of output measurements, whereas *b* is an integer that is relative to the type of sensing equipment. As mentioned before, by using the assumptions leading to Equations (96) and (97), the mass, stiffness, and damping matrices of the structural system can also be directly determined using the state-space eigenvalue matrix and the configuration-space eigenvector matrix. For this purpose, Equations (96) and (97) can be written as the following:

(102) $$U^{-1}=\Psi \Psi^{T},\;V^{-1}=\Psi \Lambda^{-1}\Psi^{T}$$

Consequently, the following algebraic equations can be deduced:

(103) $$\left[\begin{array}{cc} O & M^{-1}\\ M^{-1} & -M^{-1}RM^{-1} \end{array}\right]=\left[\begin{array}{cc} WW^{T} & W\Lambda W^{T}\\ W\Lambda W^{T} & W\Lambda^{2}W^{T} \end{array}\right]$$

and

(104) $$\left[\begin{array}{cc} -K^{-1} & O\\ O & M^{-1} \end{array}\right]=\left[\begin{array}{cc} W\Lambda^{-1}W^{T} & WW^{T}\\ WW^{T} & W\Lambda W^{T} \end{array}\right]$$

As a result, two sets of fundamental equations necessary for identifying a second-order configuration-space model from a first-order state-space representation can be written as follows:

(105) $$M^{-1}=W\Lambda W^{T},\;-M^{-1}RM^{-1}=W\Lambda^{2}W^{T}$$

and

(106) $$-K^{-1}=W\Lambda^{-1}W^{T},\;WW^{T}=O$$

Using Equations (105) and (106), the desired set of mass, stiffness, and damping matrices of the mechanical system can be directly extracted with the use of the MSD method as follows:

(107) $$\left\{\begin{array}{c} \hat{M}={\left(W\Lambda W^{T}\right)}^{-1}\\ \hat{K}=-{\left(W\Lambda^{-1}W^{T}\right)}^{-1}\\ \hat{R}=-\hat{M}W\Lambda^{2}W^{T}\hat{M} \end{array}\right.$$

where $\hat{M}$, $\hat{K}$, and $\hat{R}$ represent the identified mass matrix, with dimensions ${\hat{n}}_{x}\times {\hat{n}}_{x}$, the identified stiffness matrix, with dimensions ${\hat{n}}_{x}\times {\hat{n}}_{x}$, and the identified damping matrix, with dimensions ${\hat{n}}_{x}\times {\hat{n}}_{x}$, respectively. Equation (107) provides the basis of the MSD method for extracting second-order configuration-space models from first-order state-space representations.

### 2.8. PDC Method

The acronym PDC stands for identification of the proportional damping coefficients [97,98]. In this subsection, the PDC identification method based on a least-squares estimation process is presented for improving the identified damping matrix calculated using the MSD method. Since the identified damping matrix of the mechanical system in the MSD method is affected by the noise in the input and output signals, the identified damping matrix needs to be modified in order to obtain a more realistic configuration-space model. Assuming the proportional damping hypothesis based on the classical Rayleigh damping model, the damping matrix of the mechanical system is expressed in terms of the mass and stiffness matrices as follows:

(108) R=αM+βK

where the α and β coefficients represent the mass and stiffness proportional coefficients, respectively. The relationship between the proportional coefficients α and β with the natural angular frequencies and the damping ratios is expressed as follows:

(109) $$\xi_{i}=\frac{\alpha }{2\omega_{n,i}}+\frac{\beta \omega_{n,i}}{2},\;i=1,2,\ldots ,n_{x}$$

where $\omega_{n,i}$ and $\xi_{i}$ with $i=1,2,\ldots ,n_{x}$, respectively, denote the natural angular frequencies and the damping ratios of the mechanical system, while $n_{x}$ represents the dimension of the configuration-space model. In the PDC approach, the proportional coefficients, denoted with α and β, are estimated with a simple least-squares calculation, using the identified natural angular frequencies, indicated by ${\hat{\omega }}_{n,i}$, and the identified damping ratios, indicated by ${\hat{\xi }}_{i}$ with $i=1,2,\ldots ,{\hat{n}}_{x}$, where ${\hat{n}}_{x}$ is the number of identified degrees of freedom characterizing the identified configuration-space model. To this end, the following equations can be formulated for the mechanical system at hand:

(110) $$\bar{x}={\bar{A}}_{\omega }^{+}{\bar{b}}_{\xi }$$

where

(111) $$\bar{x}=\left[\begin{array}{c} \hat{\alpha }\\ \hat{\beta } \end{array}\right]$$

and

(112) $${\bar{A}}_{\omega }=\frac{1}{2}\left[\begin{array}{cc} \frac{1}{{\hat{\omega }}_{n,1}} & {\hat{\omega }}_{n,1}\\ \frac{1}{{\hat{\omega }}_{n,2}} & {\hat{\omega }}_{n,2}\\ \vdots & \vdots \\ \frac{1}{{\hat{\omega }}_{n,{\hat{n}}_{x}}} & {\hat{\omega }}_{n,{\hat{n}}_{x}} \end{array}\right],\;{\bar{b}}_{\xi }=\left[\begin{array}{c} {\hat{\xi }}_{1}\\ {\hat{\xi }}_{2}\\ \vdots \\ {\hat{\xi }}_{{\hat{n}}_{x}} \end{array}\right]$$

where $\bar{x}$ is a vector of unknowns, with dimensions 2×1, containing the identified proportional coefficients, denoted with $\hat{\alpha }$ and $\hat{\beta }$; while ${\bar{A}}_{\omega }$ is a coefficient matrix, having dimensions ${\hat{n}}_{x}\times 2$, and ${\bar{b}}_{\xi }$ is a right-hand-side vector having dimensions ${\hat{n}}_{x}\times 1$, constructed using the identified natural angular frequency ${\hat{\omega }}_{n,i}$ and the identified damping ratios ${\hat{\xi }}_{i}$ with $i=1,2,\ldots ,{\hat{n}}_{x}$, respectively. In Equation (110), the matrix denoted with ${\bar{A}}_{\omega }^{+}$ represents the Moore–Penrose pseudo-inverse matrix of the coefficient matrix ${\bar{A}}_{\omega }$. After the proportional coefficients are estimated with the PDC approach leading to the scalars $\hat{\alpha }$ and $\hat{\beta }$, the improved damping matrix is obtained as follows:

(113) $${\hat{R}}^{*}=\hat{\alpha }\hat{M}+\hat{\beta }\hat{K}$$

where the matrix denoted with ${\hat{R}}^{*}$ identifies the improved damping matrix with dimensions ${\hat{n}}_{x}\times {\hat{n}}_{x}$. When the mechanical system under study is lightly damped, the proportional damping matrix, denoted with ${\hat{R}}^{*}$, represents an improved estimation of the damping matrix of the mechanical system compared to the originally identified damping matrix, indicated by $\hat{R}$ and determined through the use of the MSD method.

### 2.9. MOR/BTM Method

The acronym MOR/BTM stands for the model order reduction approach combined with the balanced truncation method [99,100]. In this subsection, an MOR method combined with a BTM is described in detail for a continuous-time state-space system. The set of Lyapunov equations associated with a continuous-time state-space dynamical system can be written as follows:

(114) $$\left\{\begin{array}{c} \mathrm{AP}+PA^{T}+BB^{T}=O\\ A^{T}Q+\mathrm{QA}+C^{T}C=O \end{array}\right.$$

where O is the zero matrix with appropriate dimensions, while P and Q, respectively, represent the controllability and observability Gramian matrices, having dimensions $n_{z}\times n_{z}$, whereas A, B, and C are the continuous-time state-space matrices of the dynamical system under study, having dimensions $n_{z}\times n_{z}$, $n_{z}\times n_{u}$, and $n_{y}\times n_{z}$, respectively. The squared-root factors of the controllability and observability Gramian matrices can be written as follows:

(115) $$P=S^{T}S,\;Q=R^{T}R$$

where S and R are two matrices of dimensions $n_{z}\times n_{z}$, representing the squared-root factors of the controllability and observability Gramian matrices, respectively, denoted with P and Q. The squared-root factors can be partitioned with the use of the singular value decomposition as follows:

(116) $$SR^{T}=U\Sigma V^{T}$$

where U, Σ, and V are the matrices, all with dimensions $n_{z}\times n_{z}$, that are obtained from singular value factorization and are given by

(117) $$\Sigma =\left[\begin{array}{ccc} \Sigma_{1} & O & O\\ O & \Sigma_{2} & O\\ O & O & \Sigma_{3} \end{array}\right]$$

and

(118) $$U=\left[\begin{array}{ccc} U_{1} & U_{2} & U_{3} \end{array}\right],\;V={\left[\begin{array}{ccc} V_{1} & V_{2} & V_{3} \end{array}\right]}^{T}$$

where $n_{r}$ is the reduced dimension of the state-space model, while $U_{1}$, $U_{2}$, $U_{3}$, $V_{1}$, $V_{2}$, and $V_{3}$ represent a set of orthonormal submatrices, having, respectively, dimensions $n_{z}\times n_{z}$, $n_{z}\times n_{z}$, $n_{z}\times \left(n_{z}-2n_{r}\right)$, $n_{z}\times n_{z}$, $n_{z}\times n_{z}$, and $n_{z}\times \left(n_{z}-2n_{r}\right)$, that are obtained using singular value decomposition and contain the left singular vectors and the right singular vectors, whereas $\Sigma_{1}$, $\Sigma_{2}$, and $\Sigma_{3}$ denote three diagonal matrices, having, respectively, dimensions $n_{z}\times n_{z}$, $n_{z}\times n_{z}$, and $n_{z}\times \left(n_{z}-2n_{r}\right)$, containing the relevant singular values. Furthermore, one can write the following equations with the use of the QR-decompositions:

(119) $$S^{T}U_{i}=M_{i}X_{i},\;R^{T}V_{i}=N_{i}Y_{i}$$

where $M_{i}$ and $N_{i}$ are orthonormal matrices both of dimensions $n_{r}\times n_{r}$, while $X_{i}$ and $Y_{i}$ are non-singular matrices both of dimensions $n_{r}\times n_{r}$. The singular value decomposition of the combination of the matrices $M_{i}$ and $N_{i}$ can be written as

(120) $$N_{i}^{T}M_{i}=U_{E_{i}}\Sigma_{E_{i}}V_{E_{i}}^{T}$$

where $U_{E_{i}}$, $\Sigma_{E_{i}}$, and $V_{E_{i}}$ are three matrices, all having dimensions $n_{r}\times n_{r}$, obtained from the previous singular value factorization. With the obtained equations, a fundamental block-diagonalizing transformation matrix is determined as follows:

(121) $$Z=\left[\begin{array}{cc} Z_{1} & Z_{2} \end{array}\right]\;\Rightarrow \;Z^{+}=\left[\begin{array}{c} {\left(Z_{1}\right)}^{+}\\ {\left(Z_{2}\right)}^{+} \end{array}\right]$$

with

(122) $$\left\{\begin{array}{c} Z_{i}=V_{R_{i}}V_{E_{i}}\Sigma_{E_{i}}^{-1/2},\;\;i=1,2\\ {\left(Z_{i}\right)}^{+}=\Sigma_{E_{i}}^{-1/2}U_{E_{i}}^{T}V_{E_{i}}^{T},\;\;i=1,2 \end{array}\right.$$

where Z represents the block-diagonalizing transformation matrix of interest, having dimensions $n_{r}\times 2n_{r}$, while $Z^{+}$ represents the Moore–Penrose pseudo-inverse of the matrix Z, having dimensions $2n_{r}\times n_{r}$, whereas $Z_{1}$ and $Z_{2}$ are two submatrices, both having dimensions $n_{r}\times n_{r}$. Finally, the minimal part of the mathematical model of the dynamical system of interest can be defined with the use of the matrices Z and $Z^{+}$ as follows:

(123) $$Z^{+}\mathrm{AZ}=\left[\begin{array}{cc} {\hat{A}}_{1,1} & {\hat{A}}_{1,2}\\ {\hat{A}}_{2,1} & {\hat{A}}_{2,2} \end{array}\right]$$

and

(124) $$Z^{+}B=\left[\begin{array}{c} {\hat{B}}_{1}\\ {\hat{B}}_{2} \end{array}\right],\;\mathrm{CZ}=\left[\begin{array}{cc} {\hat{C}}_{1} & {\hat{C}}_{2} \end{array}\right]$$

where the submatrices ${\hat{A}}_{1,1}$, ${\hat{A}}_{1,2}$, ${\hat{A}}_{2,1}$, ${\hat{A}}_{2,2}$, ${\hat{B}}_{1}$, ${\hat{B}}_{2}$, ${\hat{C}}_{1}$, and ${\hat{C}}_{2}$, having, respectively, dimensions $n_{r}\times n_{r}$, $n_{r}\times n_{r}$, $n_{r}\times n_{r}$, $n_{r}\times n_{r}$, $n_{r}\times n_{u}$, $n_{r}\times n_{u}$, $n_{y}\times n_{r}$, and $n_{y}\times n_{r}$, form the desired balanced minimal state-space representation of the system dynamical model. In particular, the reduced state-space matrices of the dynamical system can be written as the following:

(125) $$\left\{\begin{array}{c} \hat{A}={\hat{A}}_{1,1}+{\hat{A}}_{1,2}{\left({\hat{A}}_{2,2}\right)}^{-1}{\hat{A}}_{2,1}\\ \hat{B}={\hat{B}}_{1}+{\hat{A}}_{1,2}{\left({\hat{A}}_{2,2}\right)}^{-1}{\hat{B}}_{2}\\ \hat{C}={\hat{C}}_{1}+{\hat{C}}_{2}{\left({\hat{A}}_{2,2}\right)}^{-1}{\hat{A}}_{2,1}\\ \hat{D}=D+{\hat{C}}_{2}{\left({\hat{A}}_{2,2}\right)}^{-1}{\hat{B}}_{2} \end{array}\right.$$

where $\hat{A}$, $\hat{B}$, $\hat{C}$, and $\hat{D}$, respectively, represent the reduced continuous-time state matrix of dimensions $n_{r}\times n_{r}$, the reduced continuous-time input influence matrix of dimensions $n_{r}\times n_{u}$, the reduced output influence matrix of dimensions $n_{y}\times n_{r}$, and the reduced direct transmission matrix of dimensions $n_{y}\times n_{u}$.

## 3. Case Study

### 3.1. Experimental Test Rig

**Figure 3:**
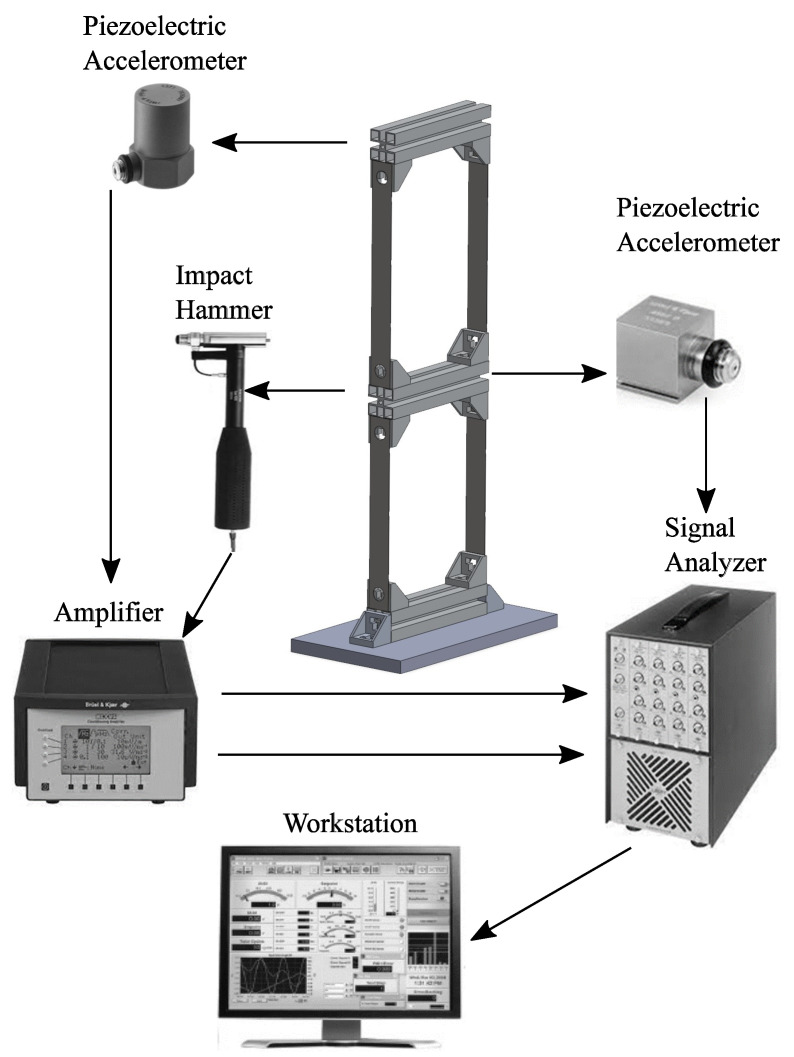
Experimental test rig for the input–output data acquisition from the flexible structure considered as the case study.

In this subsection, the description of the test rig used in the paper to obtain experimental measurements is reported. The mechanical system considered as the case study in this paper is a two-story shear building system. The structural system comprises two connecting profiles made of aluminum, which can be considered rigid rods, and four thin plates made of harmonic steel, which can be modeled as flexible beam elements. Figure 3 shows the experimental test system employed in this study.

In Figure 3, an external force serving as the input signal is applied to the structural system with a Bruel & Kjaer type 8202 impact hammer. The impact hammer consists of a rubber tip, a metallic counterweight, and a Bruel & Kjaer type 8200 load cell. The sensitivity of the impact hammer is 3.8 (pC/N). The input signal is transmitted to Bruel & Kjaer type 2692 Nexus charge amplifier, transforming the signal from 3.8 pC/N to 100 mV/N. Then, the signal is transmitted to a Bruel & Kjaer type 2825 signal analyzer.

The acceleration signals are measured as output signals for each floor of the two-story building system. The acceleration signal of the first floor is measured with a Bruel & Kjaer type 4371 piezoelectric accelerometer, having a sensitivity of 1.01 $(\mathrm{pC}\times s^{2}/m)$. The output signal is transmitted to a Bruel & Kjaer type 2635 amplifier, which transforms the output from 1.01 $(\mathrm{pC}\times s^{2}/m)$ to 10 $(\mathrm{pC}\times s^{2}/m)$, and then transmits it to the Bruel & Kjaer type 2825 signal analyzer. The acceleration signal of the second floor is measured with a Bruel & Kjaer type 4507 piezoelectric accelerometer, which has a sensitivity of 10 $(\mathrm{pC}\times s^{2}/m)$, and this experimental measurement is transmitted directly to the Bruel & Kjaer type 2825 signal analyzer.

To measure the input and output signals from the experimental system, the Bruel & Kjaer-PULSE Labshop v.4.2 software is employed. The acquisition time, denoted with $T_{s}$, is selected as equal to 32 s, while the sampling frequency, denoted with $f_{s}$, is set equal to 50 Hz, and the sampling time, denoted with Δt, is equal to 0.02 s.

### 3.2. Experimental Modal Analysis

In this subsection, the results obtained from a preliminary experimental modal analysis are reported. The outcome of this analysis serves as the reference guideline for the interpretation of the numerical and experimental results obtained from the subsequently applied system identification process, as well as for performing meaningful comparative analysis. This process is used experimentally and straightforwardly to derive the natural frequencies and damping ratios of the mechanical system at hand, and it is employed as the case study of the present investigation. For this purpose, the fast Fourier transform (FFT) is applied to the system’s time-domain vibration response to determine the experimental natural frequencies of the case study, whereas the half-power method (HPM) is employed to obtain an estimation of the damping ratios from the mechanical vibrations of the structural system.

**Figure 4:**
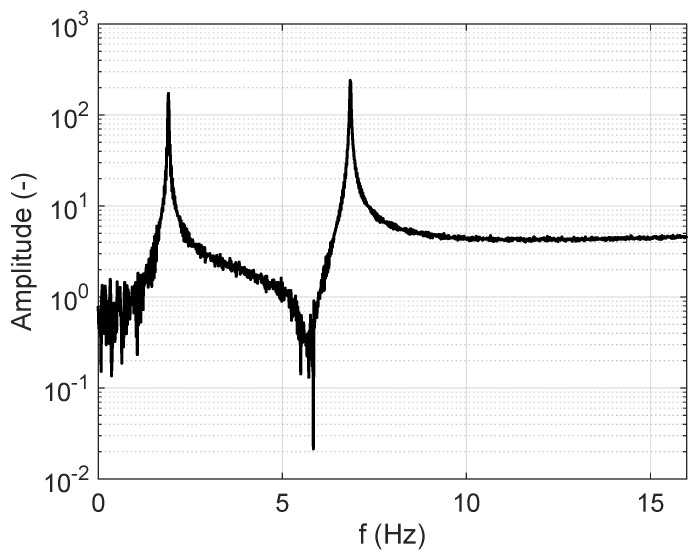
Frequency-domain experimental representation of the first floor acceleration response to the impulsive external excitation.

**Figure 8:**
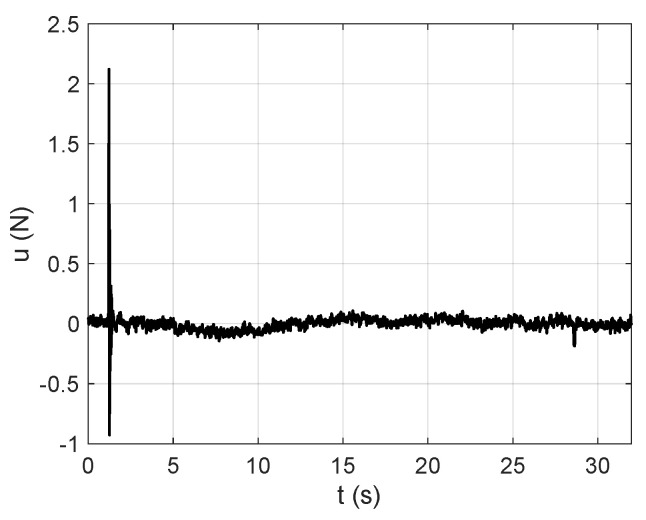
Impulsive force applied as input to the first floor of the mechanical system, recorded as the input signal.

**Figure 9:**
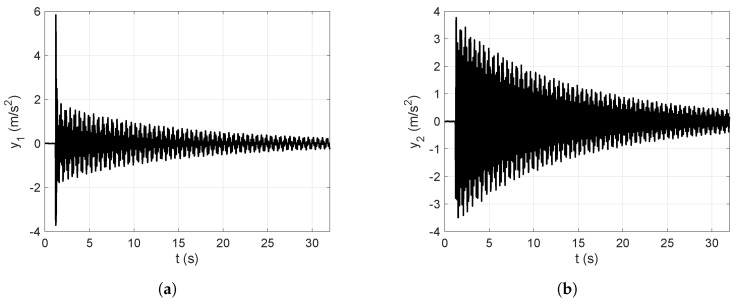
Time-domain data acquisition of the first and second output signals recorded for the two-story structural system. (**a**) First floor experimental output acceleration signal. (**b**) Second floor experimental output acceleration signal.

Figure 4 represents the frequency-domain vibration response obtained by applying the FFT to the time-domain vibration response of the first floor shown in Figure 9a, which is excited by the impulsive force signal represented in Figure 8.

**Figure 5:**
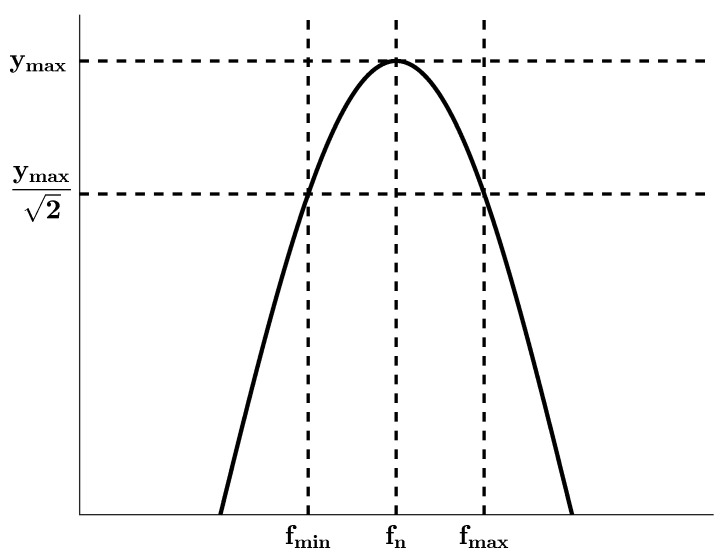
Schematic graphical representation of the application of the half-power method.

The frequency-domain signal in Figure 4 is used to experimentally obtain the first and second natural frequencies of the mechanical system of interest for this study. On the other hand, the HPM is based on finding the bandwidth for each mode to obtain the corresponding damping ratios of the system. The application of the method is geometrically demonstrated in Figure 5.

In Figure 5, $f_{n}$ and $y_{max}$, respectively, indicate the natural frequency and the maximum amplitude corresponding to the natural frequency, whereas $f_{min}$ and $f_{max}$, respectively, denote the minimum and maximum half-power frequencies. Therefore, the damping ratios of the mechanical system can be estimated with the use of the HPM as follows:

(126) $$\xi_{j}=\frac{1}{2}\frac{f_{\max,j}-f_{\min,j}}{f_{n,j}},\;j=1,2$$

where *j* is an integer corresponding to the vibration mode examined, $f_{n,j}$ and $\xi_{j}$, respectively, represent the natural frequency and the damping ratio of the system for each mode, while $f_{max,j}$ and $f_{min,j}$, respectively, identify the half-power frequencies for each mode.

The modal parameters of the mechanical system employed as the case study of this investigation, which were obtained through the experimental modal analysis discussed herein, are listed in Table 1.

### 3.3. Lumped Parameter Model of the Case Study

**Figure 6:**
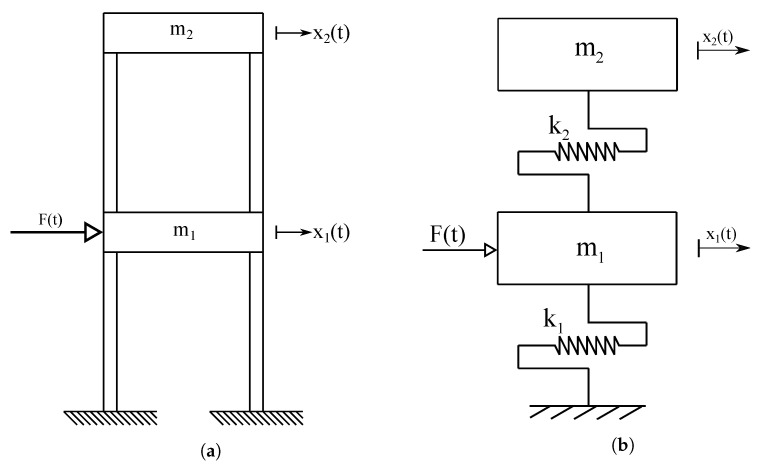
Mechanical model of the vibrating system analyzed in the paper as the case study. (**a**) Flexible structure scheme. (**b**) Lumped parameter model.

In this subsection, the lumped parameter model of the mechanical system considered as the case study is described. The lumped parameter model describes the dynamical behaviors of the two-story shear building system in a simplified way to have a preliminary assessment of its modal parameters. A schematic representation of the lumped parameter model of the mechanical system is shown in Figure 6.

In particular, Figure 6a and Figure 6b show a schematic representation of the flexible structure assumed as the case study and its lumped parameter model, respectively. The geometric and material properties of the two-story shear building system are listed in Table 2.

The lumped parameter model of the two-story shear building system includes only $n_{x}=2$ degrees of freedom. Thus, the system generalized coordinate vector is defined as follows:

(127) $$x\left(t\right)={\left[\begin{array}{cc} x_{1}\left(t\right) & x_{2}\left(t\right) \end{array}\right]}^{T}$$

where x(t) represents a time-dependent vector having dimensions $n_{x}\times 1$, whereas $x_{1}\left(t\right)$ and $x_{2}\left(t\right)$, respectively, denote the displacements of the first and second masses representing the two floors. The equations of motion of the mechanical system at hand can be written in a matrix form as follows:

(128) $$M\ddot{x}\left(t\right)+R\dot{x}\left(t\right)+\mathrm{Kx}\left(t\right)=F\left(t\right)$$

where M, R, K, and F(t), respectively, represent the system mass matrix with dimensions $n_{x}\times n_{x}$, the system damping matrix with dimensions $n_{x}\times n_{x}$, the system stiffness matrix with dimensions $n_{x}\times n_{x}$, and the system external force vector with dimensions $n_{x}\times 1$; whereas $\ddot{x}\left(t\right)$, $\dot{x}\left(t\right)$, and x(t) denote the system-generalized acceleration vector of dimensions $n_{x}\times 1$, the system-generalized velocity vector of dimensions $n_{x}\times 1$, and the system-generalized coordinate vector of dimensions $n_{x}\times 1$, respectively. The mass and stiffness matrices of the lumped parameter model of the mechanical system under study can be determined as follows:

(129) $$M=\left[\begin{array}{cc} m_{1} & 0\\ 0 & m_{2} \end{array}\right],\;K=\left[\begin{array}{cc} k_{1}+k_{2} & -k_{2}\\ -k_{2} & k_{2} \end{array}\right]$$

where $m_{1}$ and $m_{2}$, respectively, represent the first and second floor masses, whereas $k_{1}$ and $k_{2}$, respectively, denote the first and second floor stiffness. Assuming the Euler–Bernoulli beam theory, the stiffness coefficients of each flexible beam element forming the vibrating structure can be computed as follows:

(130) $$k_{1}=k_{2}=2k$$

with

(131) $$k=\frac{12EJ}{L^{3}},\;J=\frac{bh^{3}}{12}$$

where *E* is the elastic modulus of the flexible beams, *J* is the moment of area of the flexible beams, whereas *b* and *h* represent the flexible beam width and thickness, respectively.

The damping matrix of the lumped parameter model of the mechanical system can be calculated with the use of the mass and stiffness matrices considering the hypothesis of proportional damping as follows:

(132) R=αM+βK

where α and β are the first and second proportional damping coefficients. In the paper, the numerical values of the proportional damping coefficients, denoted with α and β, used for the lumped parameter model are estimated considering the metallic nature of the flexible structure analyzed as the case study.

To derive the system state-space model from its configuration-space model, a state vector of dimensions $n_{z}\times 1$ is introduced and is given by

(133) $$\begin{array}{cc} z\left(t\right) & ={\left[\begin{array}{cccc} z_{1}\left(t\right) & z_{2}\left(t\right) & z_{3}\left(t\right) & z_{4}\left(t\right) \end{array}\right]}^{T}\\ & ={\left[\begin{array}{cccc} x_{1}\left(t\right) & x_{2}\left(t\right) & {\dot{x}}_{1}\left(t\right) & {\dot{x}}_{2}\left(t\right) \end{array}\right]}^{T}\\ & ={\left[\begin{array}{cc} x^{T}\left(t\right) & {\dot{x}}^{T}\left(t\right) \end{array}\right]}^{T} \end{array}$$

where $n_{z}=2n_{x}=4$. By exploiting the definition of the state vector, one can readily derive the system’s first-order continuous-time state-space model from the second-order continuous-time configuration-space model, which can be expressed in a matrix form as follows:

(134) $$\dot{z}\left(t\right)=Az\left(t\right)+Bu\left(t\right)$$

where u(t) is the system input vector of dimensions $n_{u}\times 1$ and $n_{u}=1$ is the number of inputs. In Equation (134), the system state-space matrices that form the first-order continuous-time linear state-space model are defined as given below:

(135) $$A=\left[\begin{array}{cc} O & I\\ -M^{-1}K & -M^{-1}R \end{array}\right],\;B=\left[\begin{array}{c} O\\ M^{-1}B_{f} \end{array}\right]$$

and

(136) $$F\left(t\right)=B_{f}u\left(t\right),\;B_{f}=\left[\begin{array}{c} 1\\ 0 \end{array}\right]$$

where $B_{f}$ is the system input collocation matrix of dimensions $n_{x}\times n_{u}$ that allows for expressing the system external force vector F(t) as a linear combination of the input signals grouped in the vector u(t); A is the system state matrix, with dimensions $n_{z}\times n_{z}$; and B is the system input influence matrix, with dimensions $n_{z}\times n_{u}$; whereas O and I are appropriate zero and identity matrices, respectively.

### 3.4. Lumped Parameter Model Numerical Results

**Figure 7:**
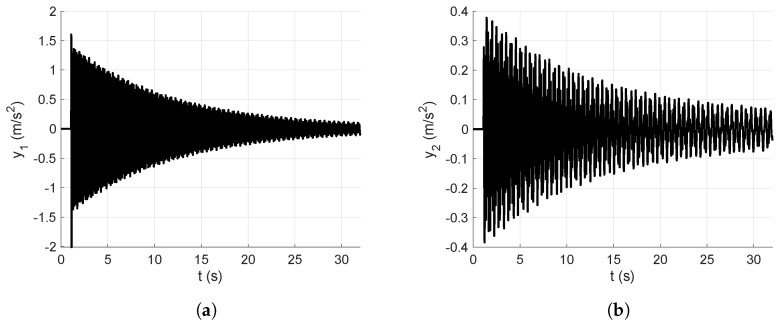
Vibration responses obtained from the lumped parameter model of the two-story mechanical system. (**a**) First floor numerical output acceleration signal. (**b**) Second floor numerical output acceleration signal.

In this subsection, the numerical vibration responses of the lumped parameter system and its modal parameters are calculated. The vibration responses are obtained from the lumped parameter model considering the input force represented in Figure 8, analyzed in Section 4. Thus, the corresponding accelerations of the first and second floors of the lumped parameter model are shown in Figure 7.

Figure 7a shows the first output acceleration obtained from the lumped parameter model, whereas Figure 7b shows the second output acceleration obtained from the lumped parameter model, both calculated through a dynamical simulation.

A modal analysis was carried out to obtain modal parameters of the lumped parameter model, such as the natural frequencies and damping ratios of the mechanical system under study. This analysis led to the set of modal parameters obtained as described below.

By performing the eigendecomposition of the lumped parameter system state matrix A, an estimation of the eigenvalues and the eigenvectors of the flexible structure can be determined, thereby leading to the following matrix factorization:

(137) $$A=\Psi \Lambda \Psi^{-1}$$

where Λ is the eigenvalue matrix, with dimensions $n_{z}\times n_{z}$, whereas Ψ is the eigenvector matrix, having dimensions $n_{z}\times n_{z}$. The mathematical forms of these matrices are defined as follows:

(138) $$\Lambda =\mathrm{diag}\left(\lambda_{1},\lambda_{2},\ldots ,\lambda_{n_{z}}\right),\;\Psi =\left[\begin{array}{cccc} \psi_{1} & \psi_{2} & \ldots & \psi_{n_{z}} \end{array}\right]$$

The natural frequencies and damping ratios of the system are then calculated using the system eigenvalues given by

(139) $$\lambda_{j}=a_{j}\pm b_{j},\;j=1,2,\ldots ,n_{z}$$

where $a_{j}$ and $b_{j}$, respectively, represent the real and imaginary parts of the eigenvalue, denoted with $\lambda_{j}$, appearing in $n_{x}=n_{z}/2$ complex conjugate pairs and associated with the complex conjugate mode shapes $\psi_{j}$ for $j=1,2,\ldots ,n_{z}$.

Finally, the system modal parameters can be calculated using the real and imaginary parts of the system eigenvalue as follows:

(140) $$f_{n,k}=\frac{\sqrt{a_{k}^{2}+b_{k}^{2}}}{2\pi },\;k=1,2,\ldots ,n_{x}$$

and

(141) $$\xi_{k}=\frac{-a_{k}}{\sqrt{a_{k}^{2}+b_{k}^{2}}},\;k=1,2,\ldots ,n_{x}$$

The natural frequencies and damping ratios obtained from the lumped parameter model of the case study are listed in Table 3.

The numerical results and the modal parameters found herein for the lumped parameter model of the case study are of remarkable importance since they serve as the guidance of the system identification process that is experimentally performed subsequently.

## 4. Results and Discussion

### 4.1. Test Rig Experimental Measurements

In this subsection, the input and output signals used for the identification of the experimental system are described. The input signal is an impulsive force recorded by using the load cell of the impact hammer, while the corresponding output signals are the acceleration signals of the first and second floors. The input and output signals measured from the experimental test rig are, respectively, shown in Figure 8 and Figure 9.

In particular, Figure 8 shows the impulse signal, considered as the input signal, whereas Figure 9a,b show the accelerations of the first and second floors obtained from the experimental test system.

### 4.2. System Identification Experimental Results

In this subsection, the experimental vibration responses are compared with the identification results obtained from the five identification methods analyzed in the paper, namely, the ARX, SSEST, N4SID, ERA/OKID, and TFEST approaches. The identified continuous-time state-space and configuration-space matrices of the flexible mechanical system considered as the case study, which were obtained by applying all the system identification techniques mentioned before to the input–output data resulting from the experimental test rig, are provided in Appendix A.

**Figure 10:**
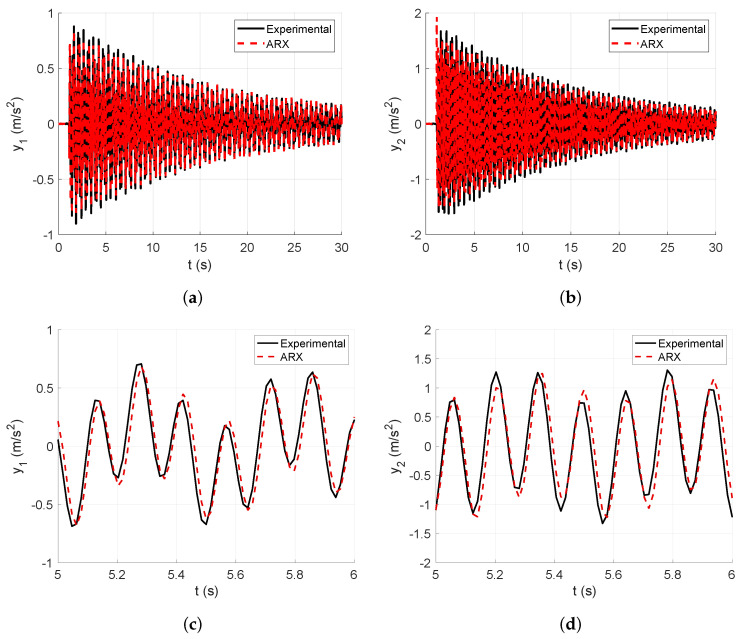
Comparison of the vibration responses obtained from the experimental test rig and the mathematical models identified by using the ARX method. (**a**) First floor acceleration. (**b**) Second floor acceleration. (**c**) First floor acceleration zoom. (**d**) Second floor acceleration zoom.

Figure 10 represents a comparison of the vibration responses of the experimental system and the identified mathematical models obtained through the use of the ARX method. In Figure 10a and Figure 10b, the vibration responses of the experimental apparatus are compared with those obtained using the identified state-space models determined with the use of the ARX method for the first and second output accelerations, respectively.

Additionally, Figure 10c and Figure 10d, respectively, represent zoomed versions of the experimental vibration responses and the identified vibration responses reconstructed employing the ARX identification method. By observing the experimental results shown in Figure 10, it is clear that the identified vibration responses obtained by using the ARX identification method successfully match the vibration response of the experimental system.

**Figure 11:**
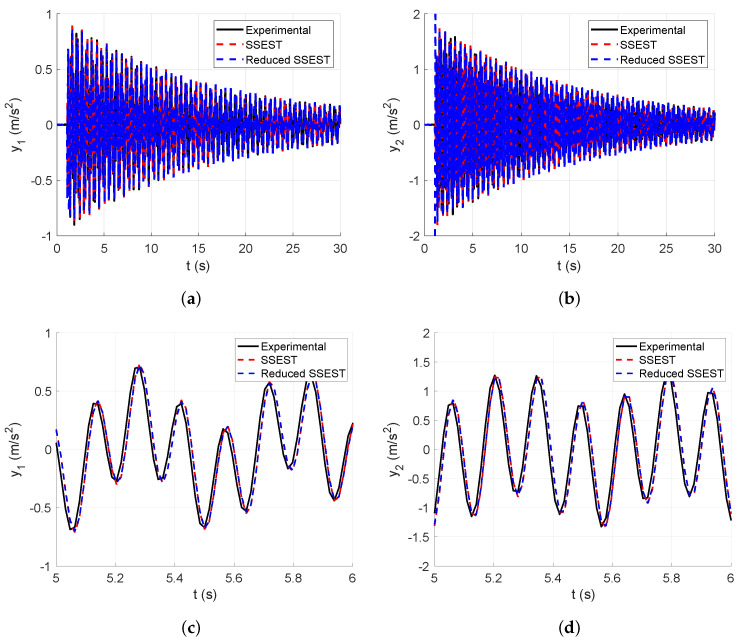
Comparison of the vibration responses obtained from the experimental test rig and the mathematical models identified by using the SSEST method. (**a**) First floor acceleration. (**b**) Second floor acceleration. (**c**) First floor acceleration zoom. (**d**) Second floor acceleration zoom.

The vibration responses of the experimental system, the dynamical response of the identified state-space models determined with the use of the SSEST method, and the dynamical response of the corresponding reduced state-space models are shown in Figure 11. In particular, in the case of the use of the SSEST identification procedure, Figure 11a represents the comparison of the time responses of the identified model obtained from the first output acceleration of the mechanical system, whereas Figure 11b represents the comparison of the time responses of the identified model obtained from the second output acceleration of the mechanical system.

Additionally, Figure 11c and Figure 11d, respectively, represent zoomed versions of the experimental vibration responses and the identified vibration responses reconstructed employing the SSEST identification method. It can be deduced from Figure 11 that the vibration responses of the experimental test rig, the identified state-space model obtained by using the SSEST identification method, and the reduced state-space model are in very good agreement.

**Figure 12:**
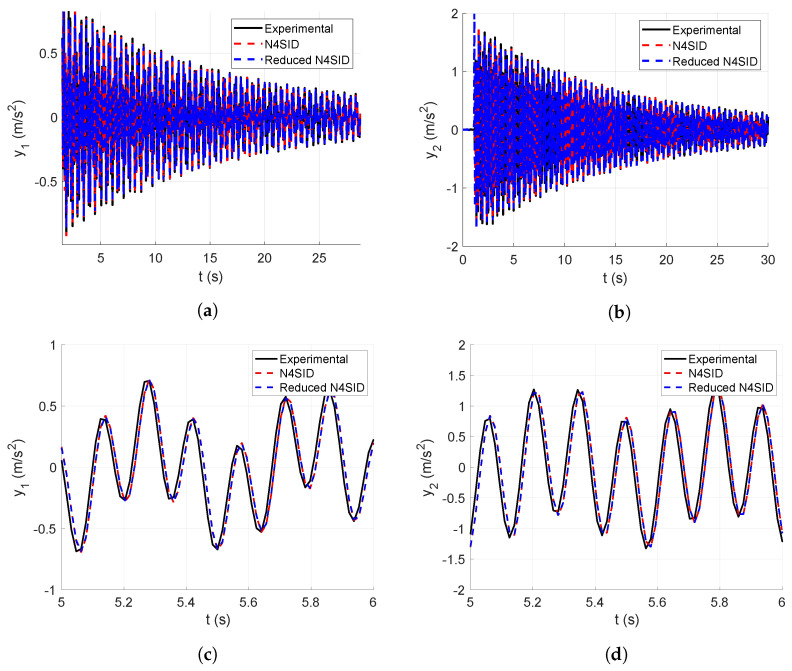
Comparison of the vibration responses obtained from the experimental test rig and the mathematical models identified by using the N4SID method. (**a**) First floor acceleration. (**b**) Second floor acceleration. (**c**) First floor acceleration zoom. (**d**) Second floor acceleration zoom.

A comparison of the vibration responses of the experimental system, the dynamical response of the identified model obtained through the use of the N4SID method, and the dynamical response of the corresponding reduced state-space models is shown in Figure 12. In Figure 12a, the vibration responses of the experimental apparatus are compared with those obtained using the identified state-space models determined with the use of the N4SID method for the first output acceleration, whereas the vibration responses are compared for the second output acceleration in Figure 12b.

Additionally, Figure 12c and Figure 12d, respectively, represent zoomed versions of the experimental vibration responses and the identified vibration responses reconstructed employing the N4SID identification method. It is apparent in Figure 12 that the vibration responses of the identified state-space model obtained by using the N4SID identification method and the reduced state-space model successfully match the vibration response of the experimental mechanical system.

**Figure 13:**
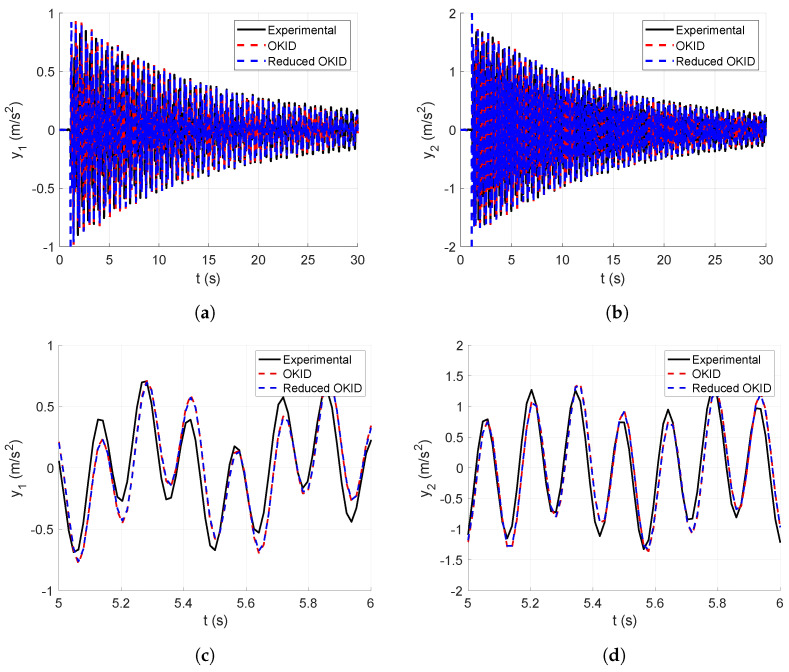
Comparison of the vibration responses obtained from the experimental test rig and the mathematical models identified by using the ERA/OKID method. (**a**) First floor acceleration. (**b**) Second floor acceleration. (**c**) First floor acceleration zoom. (**d**) Second floor acceleration zoom.

The vibration responses of the experimental test rig, the dynamical response of the identified models determined using the ERA/OKID method, and the dynamical response of the corresponding reduced continuous-time state-space models are shown in Figure 13. In Figure 13a and Figure 13b, the vibration responses of the experimental apparatus and the identified models found by employing the ERA/OKID method are compared for the first and second output accelerations, respectively.

Additionally, Figure 13c and Figure 13d, respectively, represent zoomed versions of the experimental vibration responses and the identified vibration responses reconstructed employing the ERA/OKID identification method. As shown in Figure 13, the vibration responses of the experimental system, the identified model obtained using the ERA/OKID identification method, and the reduced state-space models are in very good agreement.

**Figure 14:**
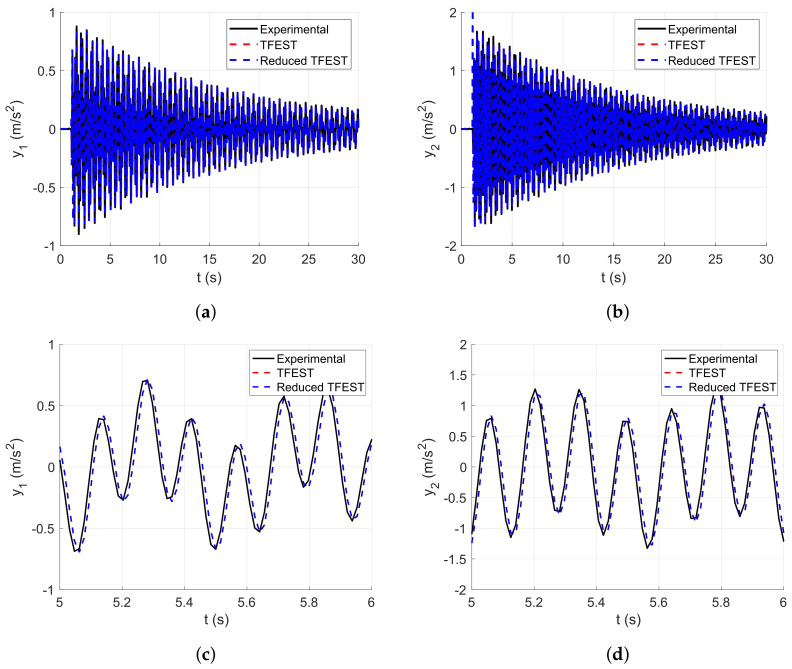
Comparison of the vibration responses obtained from the experimental test rig and the mathematical models identified by using the TFEST method. (**a**) First floor acceleration. (**b**) Second floor acceleration. (**c**) First floor acceleration zoom. (**d**) Second floor acceleration zoom.

Finally, the vibration responses of the experimental test rig, the dynamical response of the identified models determined using the TFEST method, and the dynamical response of the corresponding reduced continuous-time state-space model are shown in Figure 14. In Figure 14a and Figure 14b, the vibration responses of the experimental apparatus and the identified models deriving from the use of the TFEST method are compared for the first and second output accelerations, respectively.

Additionally, Figure 14c and Figure 14d, respectively, represent zoomed versions of the experimental vibration responses and the identified vibration responses reconstructed employing the TFEST identification method. As seen from Figure 14, the vibration responses of the experimental system, as well as the identified and the reduced state-space models obtained by using the TFEST identification method, are in very good agreement.

### 4.3. Comparative Analysis and Final Remarks

In this study, an experimental apparatus and a lumped parameter model describing the vibrating structure considered as the case study are used to test five fundamental system identification methods numerically and experimentally. The applied system identification techniques examined in this work are the ARX, SSEST, N4SID, ERA/OKID, and TFEST methods. The simulation results of the vibrating structure agree well with the vibration results obtained from the experimental system. A critical discussion on the quality of the numerical and experimental results found in this investigation is provided below.

As mentioned before, the mathematical models of the vibrating structure are estimated using the ARX, SSEST, N4SID, ERA/OKID, and TFEST system identification methods. These identification approaches were implemented in MATLAB and were individually tested using the experimental data obtained from a simple vibrating structure assumed as the case study of the paper. The mathematical model of the vibrating structure is obtained as a discrete-time transfer function in the case of the ARX method, a continuous-time state-space model in the case of the SSEST method, and discrete-time state-space models in the case of the N4SID and ERA/OKID methods, and a continuous-time transfer function in the case of the TFEST method. More specifically, different weighting matrices are studied in the analysis of the N4SID method. At the end of the preliminary analysis performed considering the weighting matrices to be used in the N4SID method, the CVA approach is chosen as the weighting matrix because this approach provides the best set of numerical results. For performing the dynamical simulations and running the system identification computational algorithms, the initial conditions are set to zero for all methods, thereby representing an initial state of rest perturbed by an impulsive external source of excitation.

To investigate the effectiveness of all the identification methods analyzed in this work, the experimental modal parameter identification problem based on the time domain is addressed and solved for the case study examined in the paper. To this end, the system modal parameters are found, encompassing the first and second natural frequencies, the first and second damping ratios, the relative errors of natural frequencies and damping ratios, and the Rayleigh damping coefficients. The set of identified values obtained from the five identified models found in this investigation is listed in Table 4.

The model orders of the identified models obtained using the five identification methods analyzed in the paper are selected by assuming the final prediction error (FPE) criterion. This criterion is defined as follows:

(142) $$\mathrm{FPE}=\frac{1+\frac{d}{N}}{1-\frac{d}{N}}\frac{1}{N}\sum_{i=1}^{N}\frac{1}{2}e_{i}^{2}$$

where *d* is the number of parameters of the identified model, *N* is the number of samples in the input and output signals of the system, e represents the identification error, and $e_{i}$ identifies the element *i* of the vector e.

The FPE criterion optimizes both the model order of the identified model and the performance of the identification process. Considering different model orders, the FPE and fit values are calculated for the five identification approaches analyzed in the paper. The model orders with high fit and low FPE values are chosen to find the optimal results from the five identification methods. More specifically, by using the FPE criteria, the state-space model order, which corresponds to the dimension of the identified state vector denoted with ${\hat{n}}_{z}$, is selected as equal to 6 for the SSEST, N4SID, and ERA/OKID methods. On the other hand, the numerator, denominator, and input/output delay orders are selected as 4, 4, and 1 for the ARX method. Differently, the numerator and denominator orders are chosen as 6 and 6 for the TFEST method. Subsequently, the MOR/BTM method is used to reduce the dimension ${\hat{n}}_{z}$ of the identified state-space models from 6 to 4 for all the system identification methods analyzed in this comparative investigation except for the ARX method.

The fitness value, denoted with fit and listed in Table 4, is employed to investigate consistency between the experimental and the identified vibration responses. For the two vibration responses, the fitness values are calculated as follows:

(143) $$\mathrm{fit}\left({\hat{y}}_{j}\right)=100\left(1-\frac{∥y_{j}-{\hat{y}}_{j}∥}{∥y_{j}-\mathrm{mean}\left(y_{j}\right)∥}\right),\;j=1,2$$

where $y_{j}$ denotes the output experimental data associated with the degree of freedom *j* and ${\hat{y}}_{j}$ represents the output identified data associated with the degree of freedom *j*, both arranged in arrays suitable for their proper computer implementation.

Another quantitative metric used to assess the quality of the results found and the performance of all the system identification methods examined in this investigation is the root mean square (RMS) deviation of the identified outputs from the experimental measurements. For this purpose, the RMS values of the experimental and identified vibration responses are calculated as follows:

(144) $$\left\{\begin{array}{c} \mathrm{RMS}(y_{j})=\sqrt{\frac{1}{n}\sum_{i=1}^{n}y_{j}^{2}\left(i\right)}\\ \mathrm{RMS}({\hat{y}}_{j})=\sqrt{\frac{1}{n}\sum_{i=1}^{n}{\hat{y}}_{j}^{2}\left(i\right)} \end{array}\right.,\;j=1,2$$

where *n* is the total number of samples, $y_{j}\left(i\right)$ is the generic sample *i* of the measured vibration signal corresponding to the degree of freedom *j*, and ${\hat{y}}_{j}\left(i\right)$ is the generic sample *i* of the identified vibration signal corresponding to the degree of freedom *j*.

The relative errors for the identified first and second natural frequencies and the identified first and second damping ratios found with each method are indeed computed by considering the corresponding experimental values. The error values for the natural frequency, damping ratio, and RMS values can be calculated as follows:

(145) $$\left\{\begin{array}{c} \mathrm{error}\left({\hat{f}}_{n,j}\right)=\left|\frac{{\hat{f}}_{n,j}-f_{n,j}}{f_{n,j}}\right|\\ \mathrm{error}\left({\hat{\xi }}_{j}\right)=\left|\frac{{\hat{\xi }}_{j}-\xi_{j}}{\xi_{j}}\right|\\ \mathrm{error}\left(\mathrm{RMS}({\hat{y}}_{j})\right)=\left|\frac{\mathrm{RMS}({\hat{y}}_{j})-\mathrm{RMS}(y_{j})}{\mathrm{RMS}(y_{j})}\right| \end{array}\right.,\;j=1,2$$

where $f_{n,j}$ and ${\hat{f}}_{n,j}$, respectively, represent the experimental and identified natural frequencies, whereas $\xi_{j}$ and ${\hat{\xi }}_{j}$ denote the experimental and identified damping ratios, respectively. Additionally, in Table 4, α and β denote the proportional damping coefficients obtained with the PDC method.

Considering the ARX, SSEST, N4SID, ERA/OKID, and TFEST identification methods one by one, it is observed from Table 4 that the fitness values are, respectively, obtained as 82.4488%, 90.4325%, 88.4624%, 69.2184%, and 86.311% for the first output, while the fitness values are, respectively, obtained as 77.6219%, 93.9063%, 86.2348%, 83.1157%, and 84.8073% for the second output. Generally, a good agreement is found when comparing the original output response and the acceleration signal calculated using all the identified models for both outputs.

According to the fitness values, the best estimation is achieved by the SSEST method, with a 90.4325% performance index for the first output and a 93.9063% performance index for the second output.

The best identification result is obtained from the TFEST method according to its relative error of the first natural frequency, equal to $1.30\times 10^{-4}$, whereas the best identification result is also obtained from the ERA/OKID method according to its relative error of the second natural frequency, equal to $9.2\times 10^{-5}$.

In the comparative analysis carried out in this work, only the predominant first two mode shapes belonging to the vibrating structure are taken into account, where indeed one has ${\hat{n}}_{z}=4$ and ${\hat{n}}_{x}=2$. Consequently, the mass, stiffness, and damping matrices obtained from the application of the MSD method are reduced to ${\hat{n}}_{x}\times {\hat{n}}_{x}$ quantities for all the identified state-space models found, as shown in Appendix A. Furthermore, the estimation of the damping matrix of the mechanical model is improved with the use of the PDC method, which relies on a simple least-squares optimization approach, starting from the identified natural frequencies and the identified damping ratios, thereby improving the quality of the identified results found in the paper by using the ARX, SSEST, N4SID, ERA/OKID, and TFEST methods.

In conclusion, in analyzing the experimental results obtained from the flexible structure considered in case study in this paper, an excellent agreement was found between the performance of all the system identification methods examined in this work. However, the principal limitation of all the system identification methods considered herein is using simultaneous input and output measurements, namely, excitation forces and vibration signals, to correctly carry out the system identification process and allow for performing experimental modal analysis. Another limitation of the approach proposed in this work that deserves clarification is the hypothesis of proportional damping; that is, the Rayleigh damping modeling. For instance, space structures tend to have high-mass modes in the low-frequency range, each with a different associated damping ratio, so they cannot generally be described by proportional damping. Nevertheless, although precise damping estimation is challenging, assuming proportional damping for lightly damped structures represents an appropriate engineering approximation since the magnitude of dissipative effects is small.

This section summarizes the applicability and limitations of the five system identification methods analyzed in this work, that is, the ARX, SSEST, N4SID, ERA/OKID, and TFEST identification techniques, providing insight into their strengths and practical challenges. Each method demonstrates robust performance under controlled experimental conditions, with excellent agreement in identifying the system dynamics of the flexible structure considered in this study. The ARX and SSEST methods are computationally efficient and straightforward for computer implementation, making them ideal for quick estimations in well-defined systems. The N4SID and ERA/OKID methods, on the other hand, excel in extracting state-space models, particularly in systems where modal properties are of interest. Finally, the TFEST method offers advantages in frequency domain analysis, providing an alternative perspective for identifying system dynamics.

Future research will be devoted to investigating several aspects and more realistic scenarios that were beyond the scope of the present study, which was based on the numerical and experimental analysis of a laboratory test rig. First, future works will analyze the family of output-only identification procedures and test these system identification algorithms for the normal modal analysis of flexible structures. Second, the performance of the identification procedures analyzed in this work will be verified in the case of mechanical systems with more than two degrees of freedom in order to validate their effectiveness. Third, different identification algorithms will be explored to reconstruct the structural matrices of flexible systems with several degrees of freedom to improve the estimation of second-order configuration-space models. Fourth, special attention will be paid to damping identification, which represents a challenging problem, especially in the presence of noise in the measurements, thereby overcoming the limitation of using the proportional damping model based on the Rayleigh hypothesis. All these highly relevant issues will be addressed and solved in future studies.

## 5. Summary, Conclusions, and Future Work

The principal research areas of the authors are multibody dynamics, nonlinear control, and system identification. In this vein, this paper is part of an extended research program focused on the interactions of these scientific disciplines. Specifically, this work represents a further step toward developing new numerical procedures for system identification of engineering interest, which are capable of reconstructing first-order state-space models and second-order configuration-space models of mechanical systems using input–output measurements describing mechanical vibrations corresponding to assigned excitation signals.

The principal objective of this paper is to provide the fundamental analytical tools and computational algorithms that form the basis of the entire research work, as well as to present the results obtained in the numerical and experimental analysis of the case study of interest for this research work by following a comparative strategy. More specifically, the numerical methods analyzed in this paper belong to the family of applied system identification methods, which are capable of constructing first-order and second-order dynamical models of a given mechanical system starting from input–output measurements. The identification methods presented in this investigation are subsequently employed to perform the experimental modal analysis of a vibrating structure considered as the case study of the paper.

This paper specifically focuses on the fundamental aspects of the principal system identification algorithms formulated in the time domain, paving the way for their subsequent experimental application to obtain dynamic models of a vibrating structure. In particular, the ARX, SSEST, N4SID, ERA/OKID, and TFEST methods are selected to estimate the mathematical model of the mechanical system of interest for this investigation. In order to obtain the first-order mechanical model of the structural system considered as the case study of the paper, the estimated discrete-time mathematical models are converted into continuous-time state-space models. Then, the mass, damping, and stiffness matrices are extracted from the continuous-time state-space models using the MSD method, and their values are normalized to compare the performance of the identification methods. Typically, the obtained damping matrices are affected by the noise of the measurement system. To improve the estimation of the damping matrix by using the PDC method, the identified natural frequencies and the identified damping ratios are used to estimate the constant coefficients that characterize the Rayleigh proportional damping model. In this way, the improved damping matrices of the mechanical system are calculated using the set of proportional coefficients, thereby improving the quality of the estimated configuration-space model describing the vibrating structure.

From a computational standpoint, in this investigation, a special-purpose computer program, developed by the authors in MATLAB, was used for implementing the ERA/OKID method, whereas the ARX, SSEST, N4SID, and TFEST methods are already implemented in the System Identification Toolbox available in MATLAB. Furthermore, while the numerical procedures based on the MOR/BTM method are already implemented in the MATLAB suite pertaining to the analysis of linear dynamical systems, both the MSD method and the PDC method were implemented by the authors in a general-purpose computer program specifically developed using MATLAB. The paper shows that improving the identified results is quite successful, especially for the ARX, SSEST, N4SID, and TFEST methods. Additionally, the experimental results show a good agreement with the simulation results, demonstrating the effectiveness and the repeatability of the identification methods considered in this investigation.

There are several interesting topics that could be addressed and studied in future works. For example, an important topic for future investigations is the experimental study of the Principal Hankel Component Algorithm (PHCA) performance for identifying first-order dynamical models of structural systems, like the simple vibrating system analyzed in this work, or considering more complex three-dimensional systems of applicative interest, such as spacecraft antennas. Another essential aspect that deserves further investigation is the reconstruction of the mass, damping, and stiffness matrices of mechanical systems starting from previously identified state-space models, such as in the case of using the technique referred to as the instrumental variable method. Finally, using the identified mechanical models to optimize control system design is an additional fundamental problem that will need to be addressed and solved in future works. All these lines of research will be followed in future investigations.

## Figures and Tables

**Table 1:** Experimental modal parameters of the mechanical structure assumed as the case study of the paper.

Symbol (Units)	Value
$f_{n,1}$ (Hz)	1.9053
$f_{n,2}$ (Hz)	6.8478
$\xi_{1}$ (-)	0.0082
$\xi_{2}$ (-)	0.0022

**Table 2:** Physical parameters of the lumped parameter model of the flexible structure assumed as the case study of the paper.

Definition	Symbol (Units)	Value
First Floor Mass	$m_{1}$ (kg)	0.65
Second Floor Mass	$m_{2}$ (kg)	1.55
Flexible Beam Thickness	*h* (m)	$1\times 10^{-3}$
Flexible Beam Width	*b* (m)	$35\times 10^{-3}$
Flexible Beam Length	*L* (m)	$300\times 10^{-3}$
Flexible Beam Elastic Modulus	*E* (N/m^2^)	$2\times 10^{11}$
First Proportional Damping Coefficient	α (1/s)	$1\times 10^{-2}$
Second Proportional Damping Coefficient	β (s)	$1\times 10^{-4}$

**Table 3:** Modal parameters of the lumped parameter model of the flexible structure assumed as the case study of the paper.

Symbol (Units)	Value
$f_{n,1}$ (Hz)	1.9487
$f_{n,2}$ (Hz)	6.7149
$\xi_{1}$ (-)	0.0010
$\xi_{2}$ (-)	0.0022

**Table 4:** Summary of the system identification experimental results obtained in this investigation for the case study of the paper.

Symbol (Units)	EXP	ARX	SSEST	N4SID	ERA/OKID	TFEST
Order (-)	-	[4, 4, 1]	6	6	6	[6, 6]
Fit $y_{1}$ (%)	-	82.4488	90.4325	88.4624	69.2184	86.311
Fit $y_{2}$ (%)	-	77.6219	93.9063	86.2348	83.1157	84.8073
$f_{n,1}$ (Hz)	1.9053	1.9089	1.9060	1.9049	1.9130	1.90555
$f_{n,2}$ (Hz)	6.8478	6.8498	6.8492	6.8504	6.8484	6.84988
$\xi_{1}$ (-)	0.0082	0.0024	0.0037	0.0039	0.0049	0.0037
$\xi_{2}$ (-)	0.0022	0.0017	0.0016	0.0016	0.0017	0.00165
RMS $y_{1}$ (m/s^2^)	0.2379	0.2356	0.2438	0.2378	0.2452	0.23894
RMS $y_{2}$ (m/s^2^)	0.4919	0.4812	0.5091	0.4998	0.4986	0.49305
Error $f_{n,1}$ (-)	-	$1.89\times 10^{-3}$	$3.54\times 10^{-4}$	$2.17\times 10^{-4}$	$4.03\times 10^{-3}$	$1.30\times 10^{-4}$
Error $f_{n,2}$ (-)	-	$2.87\times 10^{-4}$	$2.03\times 10^{-4}$	$3.75\times 10^{-4}$	$9.20\times 10^{-5}$	$3.03\times 10^{-4}$
Error $\xi_{1}$ (-)	-	$7.08\times 10^{-1}$	$5.54\times 10^{-1}$	$5.30\times 10^{-1}$	$4.08\times 10^{-1}$	$5.49\times 10^{-1}$
Error $\xi_{2}$ (-)	-	$2.26\times 10^{-1}$	$2.78\times 10^{-1}$	$2.57\times 10^{-1}$	$2.31\times 10^{-1}$	$2.52\times 10^{-1}$
Error RMS $y_{1}$ (-)	-	$9.73\times 10^{-3}$	$2.48\times 10^{-2}$	$4.75\times 10^{-4}$	$3.05\times 10^{-2}$	$4.31\times 10^{-3}$
Error RMS $y_{2}$ (-)	-	$2.18\times 10^{-2}$	$3.49\times 10^{-2}$	$1.60\times 10^{-2}$	$1.35\times 10^{-2}$	$2.28\times 10^{-3}$
α (1/s)	-	$4.98\times 10^{-2}$	$8.36\times 10^{-2}$	$8.82\times 10^{-2}$	$1.14\times 10^{-1}$	$8.40\times 10^{-2}$
β (s)	-	$5.22\times 10^{-5}$	$2.87\times 10^{-5}$	$2.83\times 10^{-5}$	$1.70\times 10^{-5}$	$3.11\times 10^{-5}$

## Data Availability

The data presented in this study are available from the corresponding author upon reasonable request.

## Appendix A. Experimental Results of All the System Identification Methods Considered in the Paper

This appendix reports the complete set of continuous-time state-space and configuration-space matrices identified using the ARX, SSEST, N4SID, ERA/OKID, and TFEST methods. More specifically, the set of matrices A, B, C, and D represent the continuous-time state-space model identified for the case study, whereas the set of matrices M, R, and K represent the corresponding configuration-space model. In the identified configuration-space model, the element $M_{1,1}$ of the identified mass matrix M was normalized to 1. For the eigenvalue analysis, the reader should refer to the state matrix A, while the structural matrices M, R, and K are reported here to demonstrate the use of the MSD method and serve different purposes.

### Appendix A.1. ARX Method Experimental Results

(A1)
$$A=\left[\begin{array}{cccc} 110.9230 & -180.9827 & 92.7214 & -22.9220\\ 22.9951 & 29.8220 & -64.4832 & 11.7559\\ -11.7934 & 64.5890 & -29.9265 & -22.9589\\ 23.0321 & -93.0249 & 181.2760 & -111.0223 \end{array}\right],\;B=\left[\begin{array}{c} -5.6956\\ -0.0026\\ 5.7094\\ -55.4421 \end{array}\right]$$

(A2)
$$C=\left[\begin{array}{cccc} -0.1752 & 0.4603 & -0.3273 & 0.0374\\ 1.0452 & -2.7122 & 2.4160 & -0.7297 \end{array}\right],\;D=\left[\begin{array}{c} 0.0527\\ -0.4463 \end{array}\right]$$

(A3)
$$M=\left[\begin{array}{cc} 1 & 1.3682\\ 1.3682 & 0.2948 \end{array}\right],\;R=\left[\begin{array}{cc} -0.0127 & -0.0455\\ -0.0455 & -0.0064 \end{array}\right],\;K=\left[\begin{array}{cc} 239.6971 & -210.9647\\ -210.9647 & 19.0061 \end{array}\right]$$

### Appendix A.2. SSEST Method Experimental Results

(A4)
$$A=\left[\begin{array}{cccc} -1.3458 & 5.1362 & 12.7376 & -6.4485\\ -4.8118 & -1.5436 & -33.5868 & -21.7159\\ -5.0074 & 29.8709 & 0.5590 & 17.7738\\ 8.6555 & 22.0790 & -20.9756 & 2.1061 \end{array}\right],\;B=\left[\begin{array}{c} -1.9499\\ -0.3642\\ -1.1048\\ 7.5953 \end{array}\right]$$

(A5)
$$C=\left[\begin{array}{cccc} 0.1023 & -0.3746 & -6.7029 & -0.9549\\ -0.1137 & -2.3234 & 11.3101 & 8.8972 \end{array}\right],\;D=\left[\begin{array}{c} 0\\ 0 \end{array}\right]$$

(A6)
$$M=\left[\begin{array}{cc} 1 & 0.3443\\ 0.3443 & 0.2443 \end{array}\right],\;R=\left[\begin{array}{cc} 0.2814 & 0.0840\\ 0.0840 & 0.0766 \end{array}\right],\;K=\left[\begin{array}{cc} 793.8613 & -175.7809\\ -175.7809 & 467.1347 \end{array}\right]$$

### Appendix A.3. N4SID Method Experimental Results

(A7)
$$A=\left[\begin{array}{cccc} -0.5468 & 14.4380 & -2.4361 & 10.1634\\ -14.3688 & 0.5026 & -1.8148 & -3.0908\\ 4.7479 & 3.7177 & -0.0905 & 24.6466\\ -19.7945 & -0.5178 & -63.7328 & -0.0982 \end{array}\right],\;B=\left[\begin{array}{c} 0.3670\\ 0.4736\\ 0.4028\\ 0.4486 \end{array}\right]$$

(A8)
$$C=\left[\begin{array}{cccc} -12.5974 & 35.1555 & -30.1865 & 2.1621\\ 29.9334 & 27.7958 & 90.5905 & -17.5409 \end{array}\right],\;D=\left[\begin{array}{c} -0.1106\\ -0.2742 \end{array}\right]$$

(A9)
$$M=\left[\begin{array}{cc} 1 & 0.2951\\ 0.2951 & 0.2431 \end{array}\right],\;R=\left[\begin{array}{cc} 0.2138 & 0.0512\\ 0.0512 & 0.0586 \end{array}\right],\;K=\left[\begin{array}{cc} 662.3066 & -225.8413\\ -225.8413 & 395.8547 \end{array}\right]$$

### Appendix A.4. ERA/OKID Method Experimental Results

(A10)
$$A=\left[\begin{array}{cccc} -1.5198 & 9.7744 & 11.2389 & 14.3809\\ -3.1692 & -0.1489 & -38.2397 & -10.4135\\ -11.1742 & 37.5630 & -0.2617 & -16.1306\\ -11.5287 & 2.0372 & 13.5554 & 1.6682 \end{array}\right],\;B=\left[\begin{array}{c} 2.0458\\ -1.8340\\ -2.7440\\ -0.3320 \end{array}\right]$$

(A11)
$$C=\left[\begin{array}{cccc} 6.0201 & -1.4977 & 6.7104 & -6.3666\\ 6.1020 & -0.2080 & -14.7772 & -16.4601 \end{array}\right],\;D=\left[\begin{array}{c} -0.0039\\ 0.0002 \end{array}\right]$$

(A12)
$$M=\left[\begin{array}{cc} 1 & 1.9622\\ 1.9622 & -0.5139 \end{array}\right],\;R=\left[\begin{array}{cc} -0.0183 & -0.0564\\ -0.0564 & 0.0189 \end{array}\right],\;K=\left[\begin{array}{cc} 390.6180 & -441.2521\\ -441.2521 & 358.5624 \end{array}\right]$$

### Appendix A.5. TFEST Method Experimental Results

(A13)
$$A=\left[\begin{array}{cccc} -0.2302 & -1995.7228 & -184.2176 & -265536.6182\\ 1 & 0 & 0 & 0\\ 0 & 1 & 0 & 0\\ 0 & 0 & 1 & 0 \end{array}\right],\;B=\left[\begin{array}{c} 1\\ 0\\ 0\\ 0 \end{array}\right]$$

(A14)
$$C=\left[\begin{array}{cccc} 0.2482 & -888.0368 & 26387.1647 & -174631.6102\\ 54.8302 & 1908.8544 & 35730.8775 & 233509.0347 \end{array}\right],\;D=\left[\begin{array}{c} 0.1095\\ 0.0015 \end{array}\right]$$

(A15)
$$M=\left[\begin{array}{cc} 1 & 0.3612\\ 0.3612 & 0.2701 \end{array}\right],\;R=\left[\begin{array}{cc} 0.2691 & 0.0840\\ 0.0840 & 0.0832 \end{array}\right],\;K=\left[\begin{array}{cc} 707.3388 & -167.9438\\ -167.9438 & 527.9694 \end{array}\right]$$
